# Proteomic Analysis of Urine Exosomes Reveals Renal Tubule Response to Leptospiral Colonization in Experimentally Infected Rats

**DOI:** 10.1371/journal.pntd.0003640

**Published:** 2015-03-20

**Authors:** Satish P. RamachandraRao, Michael A. Matthias, Chanthel-Kokoy Mondrogon, Eamon Aghania, Cathleen Park, Casey Kong, Michelle Ishaya, Assael Madrigal, Jennifer Horng, Roni Khoshaba, Anousone Bounkhoun, Fabrizio Basilico, Antonella De Palma, Anna Maria Agresta, Linda Awdishu, Robert K. Naviaux, Joseph M. Vinetz, Pierluigi Mauri

**Affiliations:** 1 University of California, San Diego Department of Medicine, Division of Nephrology-Hypertension, San Diego, California, United States of America; 2 University of California, San Diego Department of Pediatrics, Center for Promotion of Maternal Health and Infant Development, La Jolla, California, United States of America; 3 University of California, San Diego Department of Medicine, Division of Infectious Diseases, La Jolla, California, United States of America; 4 Proteomics and Metabolomics Laboratory (Istituto di Tecnologie Biomediche-Consiglio Nazionale delle Ricerche (ITB-CNR); Segrate (MI), Italy; 5 University of California, San Diego Departments of Medicine, Pediatrics and Pathology, San Diego, California, United States of America; 6 Institute of Life Sciences, Scuola Superiore Sant’Anna, Pisa, Italy; University of Tennessee, UNITED STATES

## Abstract

**Background:**

Infectious *Leptospira* colonize the kidneys of reservoir (e.g. rats) and accidental hosts such as humans. The renal response to persistent leptospiral colonization, as measured by urinary protein biosignatures, has not been systematically studied. Urinary exosomes--bioactive membrane-bound nanovesicles--contain cell-state specific cargo that additively reflect formation all along the nephron. We hypothesized that *Leptospira*-infection will alter the content of urine exosomes, and further, that these *Leptospira*-induced alterations will hold clues to unravel novel pathways related to bacterial-host interactions.

**Methodology/Principal findings:**

Exosome protein content from 24 hour urine samples of *Leptospira*-infected rats was compared with that of uninfected rats using SDS-PAGE and liquid chromatography/tandem mass spectrometry (LC-MS/MS). Statistical models were used to identify significantly dysregulated proteins in *Leptospira*-infected and uninfected rat urine exosomes. In all, 842 proteins were identified by LC-MS/MS proteomics of total rat urine and 204 proteins associated specifically with exosomes. Multivariate analysis showed that 25 proteins significantly discriminated between uninfected control and infected rats. Alanyl (membrane) aminopeptidase, also known as CD13 topped this list with the highest score, a finding we validated by Western immunoblotting. Whole urine analysis showed Tamm-Horsfall protein level reduction in the infected rat urine. Total urine and exosome proteins were significantly different in male vs. female infected rats.

**Conclusions:**

We identified exosome-associated renal tubule-specific responses to *Leptospira* infection in a rat chronic colonization model. Quantitative differences in infected male and female rat urine exosome proteins vs. uninfected controls suggest that urine exosome analysis identifies important differences in kidney function that may be of clinical and pathological significance.

## Author Summary

Leptospirosis is a bacterial disease commonly transmitted from animals to humans. Though this disease affects more than three quarters of a million people every year and takes a disproportionate toll on the poor in in tropical regions, few virulence factors have been identified and very little is known regarding the pathogenesis of leptospirosis. Symptoms vary from fever and fatigue to severe pulmonary hemorrhage and death. Approximately 5–10% of *Leptospira* infections in humans are chronic (>1 year) and asymptomatic (no overt signs of disease). Nonetheless, very little is known about the clinical significance of these infections. In this report, we show that non-invasive tools namely proteomic analysis of urinary exosomes can be used to identify differences between healthy and *Leptospira*-infected rat kidney and between *Leptospira*-infected male and female rat kidney. In future studies, these analyses will be extended to determine clinical significance and extent of renal dysfunction in the asymptomatic human.

## Introduction

Leptospirosis is among the world’s most important zoonotic infectious diseases [1,2], characterized by variable manifestations ranging from asymptomatic or self-resolving acute febrile illness to severe disease with a combination of fever, acute kidney injury, jaundice, severe pulmonary hemorrhage syndrome, refractory shock, and aseptic meningitis [3]. Important advances have been made in diverse aspects of leptospirosis including the differential host responses to leptospiral infection [4–9]. Despite these advances, mechanistic details by which end organ damage develops in some individuals but not in others remain to be elucidated. Further, factors that govern host susceptibility to leptospiral infection are not well understood. A recent study by our collaborative group found that approximately 6% of randomly sampled individuals in a highly endemic rural Amazonian village were chronically colonized by *Leptospira* without any recent clinical evidence of infection [10]. Although renal colonization by leptospires may occur in humans without serological or clinical evidence of infection, the clinical relevance and functional consequences of leptospiral colonization in humans remain to be characterized. New, less invasive, less expensive and more practical tools (compared to kidney biopsy) are needed to study pathologic changes in the kidney in order to understand clinically relevant sequelae of infection. In this report, we use proteomic analysis of urine exosomes in a rat chronic colonization model as a non-invasive window to kidney function in asymptomatic leptospiral infection.

Exosomes are nanovesicles that are released from cells as a mechanism of intercellular communication [11]. Characterization of exosomes from different biological samples has shown the presence of common as well as cell-type specific proteins. The protein content of exosomes has been shown to be modified under pathological or stress conditions [12–14]. Since exosome contents are specifically derived from cellular components, here we tested the hypothesis that urinary exosome protein content from a rat infected with *Leptospira* would be different from that of the uninfected rat, and that these differences would hold key information about the pathways mediating host responses to *Leptospira* infection.

After renal colonization, persistent shedding of *Leptospira* is clearly established in carrier animal hosts, especially rodents. However, they rarely develop symptoms and are not noticeably impaired by infection of their kidneys [1,15,16]. We have recently detected chronic asymptomatic renal colonization by *Leptospira* in human subjects from a rural Amazonian village [10]. We reasoned that the structural and functional changes in the kidney that arise following asymptomatic *Leptospira* infection are different from symptomatic disease. These differences between the asymptomatic and symptomatic leptospirotic kidneys can be understood by studying the downstream products of the kidney, such as urine.

Given the nephron cell-state-specific cargo of the urinary exosome, we hypothesized that urine exosome analysis holds key information that is relevant to differences between clinically symptomatic and asymptomatic leptospirosis infection. This report is the first step towards testing this hypothesis. Here we report our preliminary findings from the exosome proteomic analyses of urines from rats infected with *Leptospira* using uninfected rat urine exosome as controls. We also studied the host-response to *Leptospira* infection in male and female rats separately.

## Materials and Methods

### Ethics statement

This work was approved by the Institutional Animal Care and Use Committee of the University of California, San Diego.

### Experimental infection of animals and study design

Three-week old Sprague Dawley rats (Charles River Laboratories, USA) (6 male rats and 6 female rats) were housed in cages of 1–2 rats each, with food and water provided *ad libidum*. Six animals (3 males and 3 females) were inoculated intraperitoneally with 10^8^ mid-log phase *L*. *interrogans* serovar **Copenhageni** strain HAI1026. Uninfected controls were inoculated ip with sterile EMJH. To determine the health status, weight, general body condition, posture, activity, appetite food and water consumption of each animal was monitored daily. Infection was confirmed by serology (microscopic agglutination test) and quantitative polymerase chain reaction (qPCR) of weekly urines, and Warthin-Starry silver staining of kidney sections following necropsy. In order to keep the minimum number of animals for the purpose of statistical calculations, we used this small number of animals. This is also discussed as a limitation of the study under discussion.

### Urine sampling and processing

Urine from each animal was collected weekly starting 7 days after post-challenge by placing the animals individually for 20–24 hrs in metabolic cages, and was captured into containers containing Roche Complete Protease Inhibitor, one tablet per 5 mL urine. Urines were separately centrifuged at 3000 x *g* for 30 min. The supernatant was withdrawn, the pH adjusted to 7, aliquoted and frozen at -70°C until further analysis.

Genomic DNA was extracted from these weekly urines and qPCR was used to assess leptospiruria. In addition, at necropsy, kidneys were harvested, fixed in formalin, paraffin embedded, and infection confirmed by Warthin-Starry silver stain (S1 and S2 Figs.).

### Exosome preparation and protein resolution analysis

Exosomes from terminal urine samples from infected and uninfected rats were prepared using an in-house protocol developed based on the solvent exclusion principle using polyethylene glycol (PEG)-induced precipitation. To prevent naturally occurring peptides in the exosome from confounding post in-gel trypsinization peptide information of the full-length proteins, we conducted 1 dimensional SDS-PAGE of the exosome proteins prior to in-gel trypsinization.

### Proteomic analysis

Each rat urine sample (only terminal sample) was separately analyzed, without pooling any sample. Each rat urine sample was run in a separate gel lane. Gel slices for each lane were cut to 1 mm x 1 mm cubes and destained 3 times by first washing with 100 *μL* of 100 mM ammonium bicarbonate for 15 min, followed by addition of the same volume of acetonitrile (ACN) for 15 min [17]. The supernatant was transferred to a clean tube and samples lyophilized and reduced by mixing with 200 *μ*L of 100 mM ammonium bicarbonate-10 mM DTT then incubated at 56°C for 30 min. The liquid was removed and 200 *μ*L of 100 mM ammonium bicarbonate-55mM **iodoacetamide** was added to gel pieces, which were then incubated at room temperature in the dark for 20 min. After removal of the supernatant and one wash with 100 mM ammonium bicarbonate for 15 min, an equal volume of ACN was added to dehydrate the gel pieces. The solution was then removed and samples lyophilized.

For digestion, ice-cold trypsin (0.01 *μ*g/*μ*L) in 50 mM ammonium bicarbonate solution was added in enough amounts to cover the gel pieces and set on ice for 30 min. After complete rehydration, the excess trypsin solution was removed, replaced with fresh 50 mM ammonium bicarbonate, and left overnight at 37°C. The peptides were extracted twice by the addition of 50 *μ*l of 0.2% formic acid and 5% ACN and vortexed at room temperature for 30 min. The supernatant was removed and saved. A total of 50 *μ*L of 50% ACN-0.2% formic acid was added to the sample, which was vortexed again at room temperature for 30 min. The supernatant was removed and combined with the supernatant from the first extraction. The combined extractions from all the gel slices in a lane pertaining to a single rat urine exosome sample was separately analyzed directly by liquid chromatography (LC) in combination with tandem mass spectroscopy (MS/MS) using electrospray ionization. Thus, multiple slices of gels representing a single rat urine exosome sample was used for mass spectrometry. Two replicates per rat exosome sample were run on the MS.

Trypsin-digested mixtures were analyzed by the Eksigent nanoLC-Ultra 2D System (Eksigent, AB SCIEX Dublin, CA, USA) combined with cHiPLC-nanoflex system (Eksigent) in trap-elute mode. Briefly, samples were first loaded on the cHiPLC trap (200 μm x 500 μm ChromXP C18-CL, 3 μm, 120 Å) and washed in isocratic mode with 0.1% aqueous formic acid for 10 min at a flow rate of 3 μL/min. The automatic switching of cHiPLC ten-port valve then eluted the trapped mixture on a nano cHiPLC column (75 μm x 15 cm ChromXP C18-CL, 3 μm, 120 Å), through a 45 min gradient of 5–50% of eluent B (eluent A, 0.1% formic acid in water; eluent B, 0.1% formic acid in acetonitrile), at a flow rate of 300 nL/min. To preserve system stability, in terms of elution times of components, trap and column were maintained at 35°C.

Mass spectra were acquired using a QExactive mass spectrometer (Thermo Fisher Scientific, San José, CA, USA), equipped with a nanospray ionization source (Thermo Fisher). Nanospray was achieved using a coated fused silica emitter (New Objective, Woburn, MA, USA) (360 μm o.d./50 μm i.d.; 730 μm tip i.d.) held at 1.5 kV. The ion transfer capillary was held at 220°C. Full mass spectra were recorded in positive ion mode over a 400–1600 m/z range and with a resolution setting of 70000 FWHM (@ m/z 200) with 1 microscan per sec. Each full scan was followed by 7 MS/MS events, acquired at a resolution of 17,500 FWHM, sequentially generated in a data dependent manner on the top seven most abundant isotope patterns with charge ≥2, selected with an isolation window of 2 m/z for the survey scan, fragmented by higher energy collisional dissociation (HCD) with normalized collision energies of 30 and dynamically excluded for 30 s. The maximum ion injection times for the survey scan and the MS/MS scans were 50 and 200 ms and the ion target values were set at 10^6^ and 10^5^, respectively.

### Data management

All data generated were searched using the Sequest search engine contained in the Thermo Scientific Proteome Discoverer software, version 1.4. The experimental MS/MS spectra were correlated to tryptic peptide sequences by comparison with the theoretical mass spectra obtained by *in silico* digestion of the R*attus norvegicus* protein database downloaded January 2013 from the National Centre for Biotechnology Information (NCBI) website (www.ncbi.nlm.nih.gov). The following criteria were used for the identification of peptide sequences and related proteins: trypsin as enzyme; three missed cleavages per peptide were allowed and mass tolerances of ± 50 ppm for precursor ions and ± 0.8 Da for fragment ions were used. Validation based on separate target and decoy searches and subsequent calculation of classical score-based false discovery rates (FDR) were used for assessing the statistical significance of the identifications. Finally, to assign a final score to proteins, the SEQUEST output data were filtered as follows: 1,5; 2.0; 2.25 and 2.5 were chosen as minimum values of correlation score (Xcorr) for single-; double-; triple- and quadrupole-charged ions, respectively. A high stringency was guaranteed using parameters previously described [18] and the false-positive peptide ratio, calculated through a reverse database, was less than 3%. The output data, protein lists, obtained from the Sequest search were compared using an in-house software, namely, the Multidimensional Algorithm Protein Map (MAProMa) [19].

### Normalized spectral abundance factor of the identified urine exosome proteins

The relative abundance of polypeptides was calculated from the normalized spectral abundance factor (NSAF) using the method of Paoletti et al [20] taking into consideration the number of peptides as well as the length of the polypeptide contributing to their respective abundance. To enable comparison of samples from a subject across different time points or across different groups, each animal’s total proteome was normalized to 1. Subsequently the relative contribution of each protein from a given animal from a group was expressed as percentage of the total. Peptide numbers corresponding to a protein were thus more of raw data nature whereas the NSAF number included the peptide number as well as the total length of the protein. The peptide counts data were log-transformed prior to analysis by multivariate partial least squares discriminant analysis (PLSDA), and univariate 1-way ANOVA with unpaired comparisons, Variable Importance in Projection analysis and post hoc correction by Wilcoxon Rank test in MetaboAnalyst [21].

### Data processing and statistical analysis

In all analyses, *p* ≤ 0.05 was considered statistically significant. Analyses including Student’s t-tests, Partial-Least Squares Discriminant Analysis (PLS-DA) and variable importance in projection (VIP) were performed with the MetaboAnalyst 2.0 web portal (www.metaboanalyst.ca) [21]. To reduce systematic variance and to improve the performance for downstream statistical analysis normalization and transformation of raw data were performed before the t-tests, PLS-DA and VIP analysis. Normalization by sum of the spectral count as mentioned previously was used to overcome the variance between the analyzed samples. To make each feature comparable in magnitude to each other, data were transformed by taking the natural log of the concentration values of the analyzed proteins. The data were additionally auto-scaled (mean-centered and divided by the standard deviation of each variable).

Univariate analysis was used to check the differences in the concentrations of the analyzed exosome protein spectral count between the control and infected rat urine samples. The infected rat urines were also split into male rat and female rat categories. Paired Student’s t-test was applied to examine each variable (ratio of individual protein to total concentration in each group considered).

PLS-DA and VIP were used both for the classification and significant feature selection. A VIP plot, which is commonly used in PLS-DA, ranks proteins based on their importance in discrimination between the urinary exosomes from infected and the uninfected rats. The VIP score is a weighted sum of squares of the PLS loadings. The amount of explained Y-variance in each dimension influenced the weights [22]. Protein candidates with a false discovery rate (FDR) of ≤10% were qualified for subsequent validation by Western immunoblotting.

### Western immunoblotting and quantification

Antibody against alanyl aminopeptidase was purchased from Proteintech Group, Inc., (Chicago, IL, USA). The THP antibody was from Sigma Chemical Co (St. Louis, MO, USA). HRP-conjugated secondary antibody was from GE Life Sciences (Piscataway, NJ, USA). SDS-PAGE gels (with 10% acrylamide) were used to resolve 100 μg of protein either from exosomes or total urines of male and female rats infected with *L*. *interrogans* serovar Copenhageni. After separation the proteins were transferred to nitrocellulose paper, blocked, and incubated with primary antibody overnight before washing with Tris-buffered saline, incubation for 1 h with HRP-secondary antibody conjugate and visualized by developing as described in previous publications from our laboratory [23,24]. The quantification of the Western immunoblot bands was performed using Image J software (NIH) as previously described [24], and plotted using Graphpad Prism software (San Diego, CA, USA).

## Results

### Urine protein content is different in Lepto-infected and uninfected control rats

**Fig 1:**
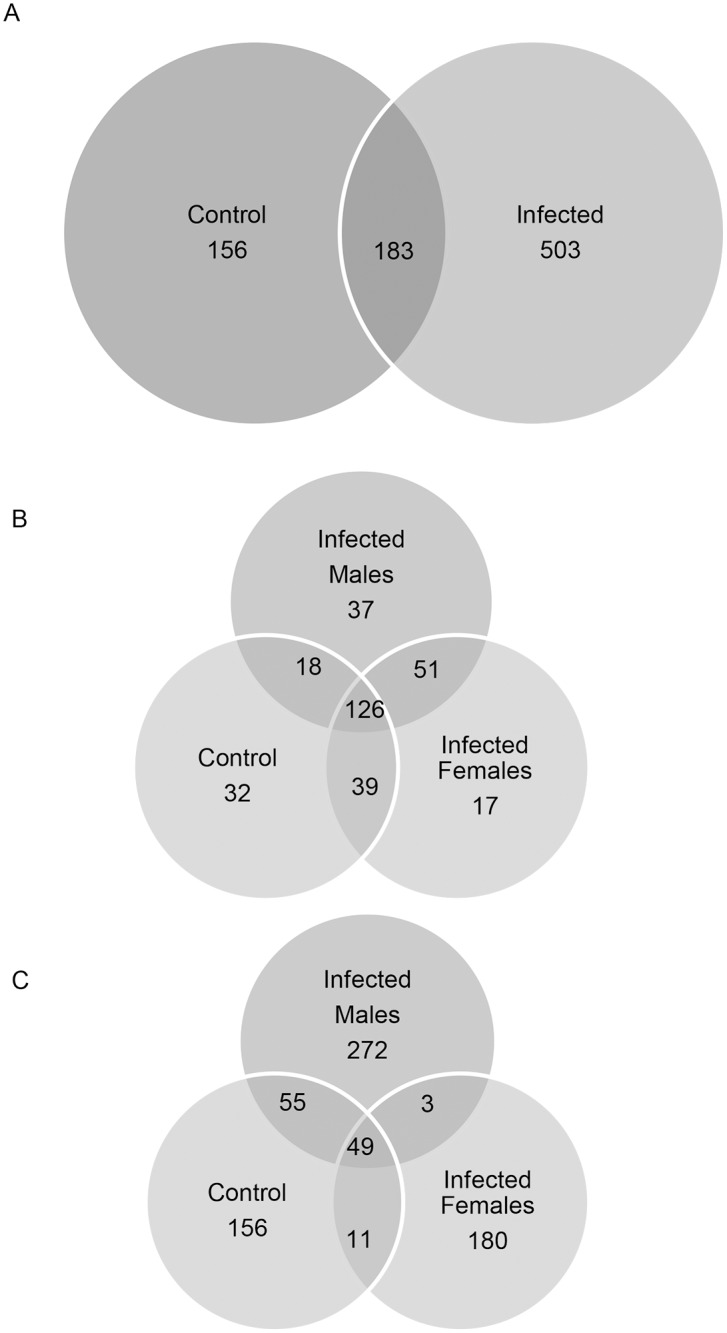
Panel A: Venn diagram summarizing the total urine proteomics data. A total of 842 proteins were identified by LC/MS-MS of the rat urines. The *Leptospira*-infected and uninfected control rat urine samples were used for generating this 2-way split between control rats and infected rats. Panel B: Venn diagram summarizing the total urine proteomics data. The *Leptospira*-infected male, female and uninfected control rat urine samples were used for generating this 3-way split among control rats, infected male and infected female rats. Panel C: Venn diagram summarizing the urine exosome proteomics data. A total of 204 proteins were identified by LC/MS-MS of the rat urine exosome proteins resolved by 1-d gel electrophoresis and trypsinization of the gel slices.

Rat urine samples were analyzed for overall protein identification by a combination of SDS-PAGE and mass-spectrometry. We found that the infected rat urine shows an overwhelming increase in both quality and quantity of the protein content, as shown in Panel A of Fig. 1 (156 versus 503 proteins unique to uninfected versus infected urines). A total of 842 proteins were detected, with further classification of subgroups in the infected animals based on gender as shown in Panel B of Fig. 1. In total, 842 proteins were identified from the total urine animals with distribution as shown in Fig. 1 Panel A and B). Importantly, 180 proteins and 272 proteins were unique to the urines of female and male rats infected with *L*. *interrogans* serovar Copenhageni respectively, as compared to 156 proteins unique to the uninfected rat urines. These differences in the composition and quantity of proteins from infected and uninfected rats potentially indicates the reactions induced in these animals by leptospiral infection. The identity of each of these proteins is as given in S1 Table.

### Urine exosomes from Leptospira-colonized rats show different protein constitution compared to uninfected control rats

Given that the urine exosomes reflect intracellular milieu of all types of various cells lining the nephron in kidney, and emerging evidence from literature that kidney functional alterations are induced due to leptospiral infection, we next focused on exosomes. We found that a total of 204 exosome proteins were identified classifiable into 7 different groups as noted in S2a-S2g Table and summarized in the Venn diagram (Panel C, Fig. 1). The exosome protein constitution also showed increase in the number of proteins expressed in the exosome, viz 32 proteins uniquely present in the uninfected exosome versus 57 unique proteins in the infected rat exosome.

**Fig 2:**
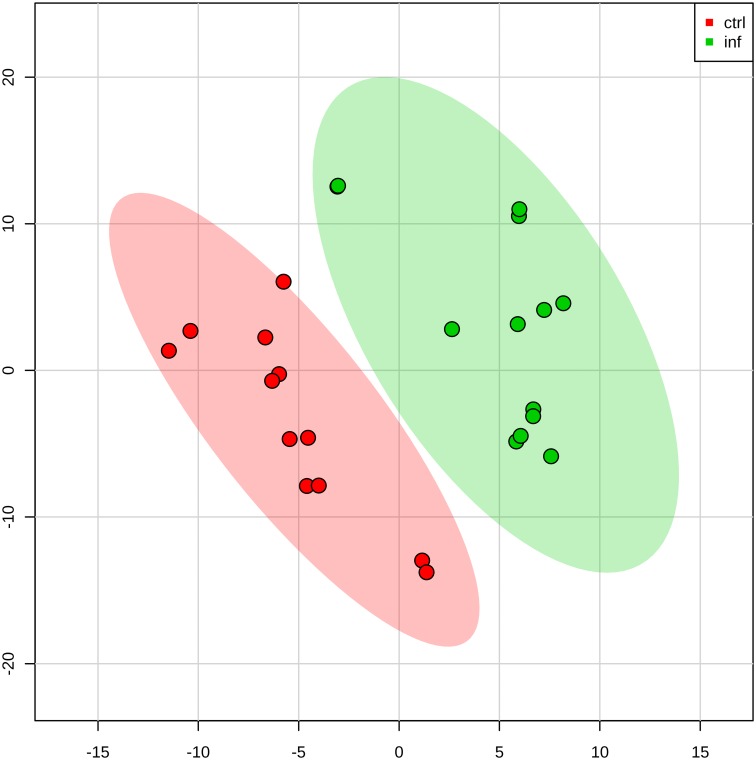
Two-dimensional (2D) partial least squares discriminant analysis separation using protein peptide count concentration-based proteomic measurements in the urine exosome of rats infected with *Leptospira* vs control rats without *Leptospira* infection (n = 3). Clear separation of rat urine exosome proteins for control versus infected is observed, signified by the lack of overlap between the two groups of exosome proteins.

We further conducted the multivariate partial least squares-discriminant analysis (PLS-DA) on these proteins. The analysis depicted in Fig. 2 shows clear separation between uninfected and infected rat urine exosome protein content, suggesting the differences between uninfected and infected rats.

### Sex-specific alteration of the rat urine exosome protein content in relation to leptospiral colonization

**Fig 3:**
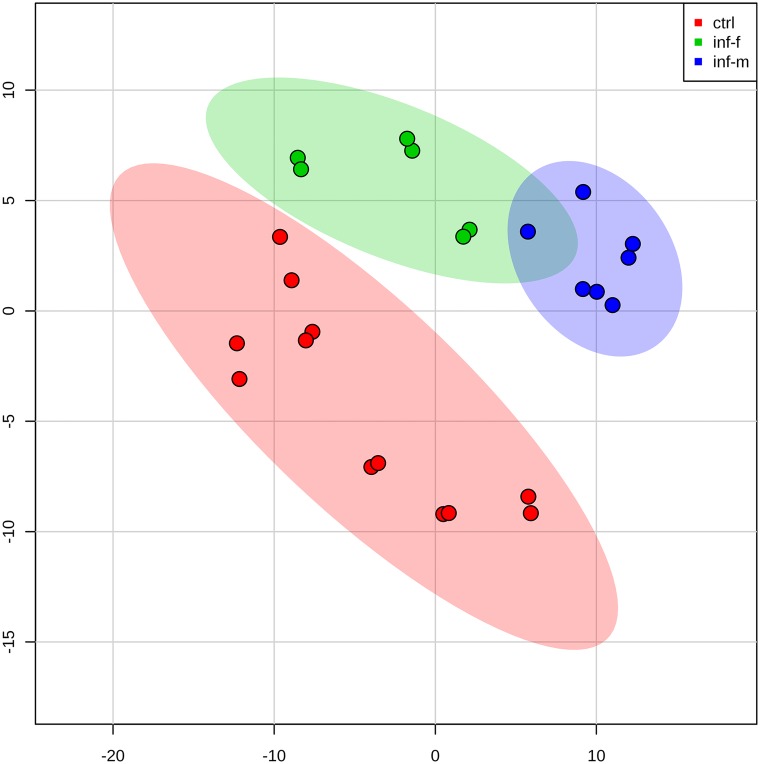
Two-dimensional (2D) partial least squares discriminant analysis separation using peptide count concentration-based proteomic measurements in the urine exosome of the rats infected with *Leptospira* vs control rats without *Leptospira* infection (n = 3). Although clear separation between control and infected rat urine exosomes is seen, a fraction of overlap between the infected male and infected female rat urine exosome proteins is observed.

**Fig 4:**
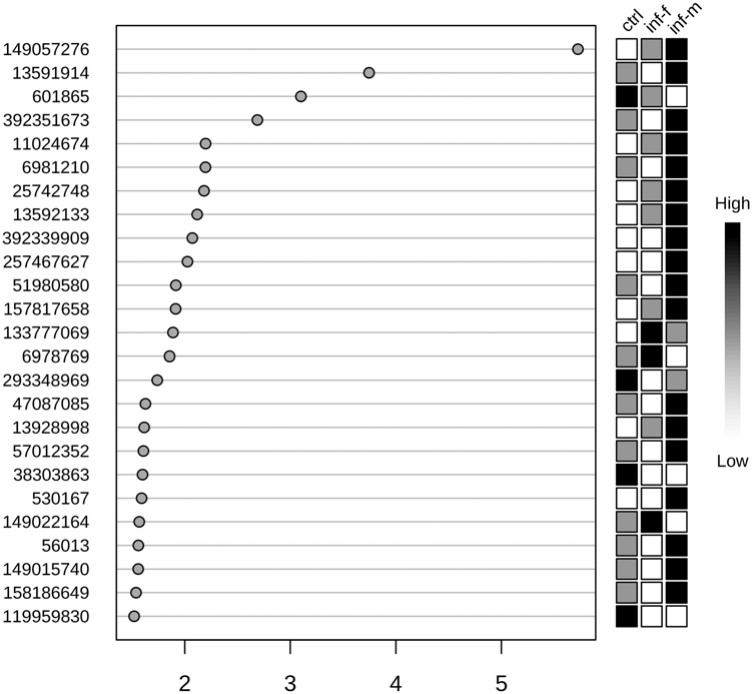
Variable importance in projection (VIP) plot: important features (analyzed serum free amino acids) identified by PLS-DA in a descending order of importance. The graph represents relative contribution of proteins to the variance between the *Leptospira*-infected and uninfected control rat urine exosomes. High value of VIP score indicates great contribution of the proteins to the group separation. The green and red boxes on the right indicate whether the protein concentration is increased (green) or decreased (red) in the exosome of the infected rat urine vs. uninfected rat urine samples. For higher n value, a VIP score of 1.5 is considered to enable discrimination between 2 phenotypes. Even with the low n (= 3) per group that is employed in this study, the VIP score of the top 3 proteins is higher than 3, increasing the confidence. Alanyl (membrane) aminopeptidase, also called CD13 is the top protein with a VIP score of 5.72.

Urinary exosome proteins in male vs. female infected rat urine were different as determined by PLS-discriminant analysis (Fig. 3). Moreover, the infected male rat urine exosome contents were far more different from those of uninfected rats compared to the infected female rat urine exosome contents. The VIP (**V**ariable **I**mportance in **P**rojection) score of 25 proteins was higher than 1.5 (Fig. 4 and Table 1). Qualitatively, a total of 57 proteins were present among all the infected rats. Of these, only 3 were shared between infected males and females, while 37 were unique to infected males and 17 were unique to infected females. Further, we conducted separate analyses of proteins between proteins of proteins of male infected and female infected rats. Table 2 depicts male infected versus control rat urine exosome analysis. Accordingly, 11 proteins were significantly altered (*p* < 0.05). Table 3 depicts female infected versus control rat urine exoosme analysis, according to which the number of significantly dysregulated proteins was 9. In the male infected rat urine exosome, the alanyl (membrane) aminopeptidase upregulation not only reached the highest level of significance (*p* = 0.00019) but also had the lower FDR (3.22%). In the female infected rat however, although this upregulation was significant compared to the uninfected rat, the FDR did not reach the cutoff of <10% (14.37%). Thus both qualitatively and quantitatively, the protein content of exosomes showed gender specificity in infected rats.

**Table 1:** Top discriminators between control, infected male & infected female rat urine exosome proteins.

GI #	VIP score	Protein Identification	Serial #
149057276	5.7252	alanyl (membrane) aminopeptidase	**VIP1**
13591914	3.7462	aminopeptidase N precursor	**VIP2**
601865	3.1009	aminopeptidase M	**VIP3**
392351673	2.6881	PREDICTED: LOW QUALITY PROTEIN: uncharacterized protein LOC287750	**VIP4**
11024674	2.1973	Na(+)/H(+) exchange regulatory cofactor NHE-RF1	**VIP5**
6981210	2.1961	Neprilysin	**VIP6**
25742748	2.1827	glutamate—cysteine ligase catalytic subunit	**VIP7**
13592133	2.1177	actin, cytoplasmic 1	**VIP8**
392339909	2.0721	PREDICTED: uncharacterized protein LOC679818	**VIP9**
257467627	2.0255	Fc fragment of IgG binding protein-like precursor	**VIP10**
51980580	1.9156	Meprin 1 beta	**VIP11**
157817658	1.9135	villin-1	**VIP12**
133777069	1.8884	Serine (or cysteine) peptidase inhibitor, clade A, member 3K	**VIP13**
6978769	1.8561	deoxyribonuclease-1 precursor	**VIP14**
293348969	1.7392	PREDICTED: keratin, type II cytoskeletal 6A-like	**VIP15**
47087085	1.6273	keratin, type I cytoskeletal 17	**VIP16**
13928998	1.6155	Na(+)/H(+) exchange regulatory cofactor NHE-RF3	**VIP17**
57012352	1.6082	keratin, type II cytoskeletal 75	**VIP18**
38303863	1.5999	Egf protein	**VIP19**
530167	1.5913	ventral prostate-specific protein	**VIP20**
149022164	1.5687	rCG26871, isoform CRA_c	**VIP21**
56013	1.5602	CRP2	**VIP22**
149015740	1.5588	rCG39189, isoform CRA_b	**VIP23**
158186649	1.5384	alpha-enolase	**VIP24**
119959830	1.5203	beta-actin	**VIP25**

**Table 2:** Male infected versus Control.

sl #	gi #	p.value	FDR	% FDR	protein identity
1	149057276	0.00019	0.032231	3.2231	alanyl (membrane) aminopeptidase
2	13592133	0.000636	0.053409	5.3409	actin, cytoplasmic 1
3	133777069	0.000943	0.053409	5.3409	Serine (or cysteine) peptidase inhibitor, clade A, member 3K
4	32563565	0.024998	0.60267	60.267	Serine protease inhibitor
5	392339909	0.025984	0.60267	60.267	PREDICTED: uncharacterized protein LOC679818
6	257467627	0.027061	0.60267	60.267	Fc fragment of IgG binding protein-like precursor
7	149028871	0.029327	0.60267	60.267	annexin A2, isoform CRA_b
8	89573967	0.031305	0.60267	60.267	isocitrate dehydrogenase 1
9	10198602	0.032149	0.60267	60.267	collectrin precursor
10	392354923	0.037988	0.60267	60.267	PREDICTED: glyceraldehyde-3-phosphate dehydrogenase-like
11	149061924	0.041715	0.60267	60.267	rCG48611, isoform CRA_c

Proteins significantly dysregulated between exosomes from urines of control and male infected rats.

**Table 3:** Female infected versus Control.

sl #	gi #	p.value	FDR	% FDR	protein identity
1	58865770	0.0001	0.00983	0.9837	solute carrier family 7 member 13
2	13592133	0.010362	0.1437	14.37	actin, cytoplasmic 1
3	149057276	0.010425	0.1437	14.37	alanyl (membrane) aminopeptidase
4	57012358	0.010506	0.1437	14.37	keratin, type II cytoskeletal 73
5	11024674	0.010675	0.1437	14.37	Na(+)/H(+) exchange regulatory cofactor NHE-RF1
6	57231	0.010759	0.1437	14.37	unnamed protein product
7	119959830	0.01163	0.1437	14.37	beta-actin
8	25742748	0.01173	0.1437	14.37	glutamate—cysteine ligase catalytic subunit
9	347800746	0.049478	0.34739	34.739	serine protease inhibitor A3K precursor

Proteins significantly dysregulated between exosomes from urines of control and female infected rats.

Of interest, the top discriminator between control and infected rats, namely alanyl aminopeptidase of the membrane origin,is also known as **a**mino**p**eptidase **n**eutral (APN) or CD13 [25,26]. Furthermore, CD13 also shows different levels of dysregulation between infected male and infected female rats (Table 1).

### The urinary exosome membrane alanyl aminopeptidase or CD13 is a marker of infection

A separate analysis of the proteins dysregulated between control and infected rats showed that 11 proteins were significantly different in urinary exosomes (*p* <0.05, Table 4). However, only one protein had an FDR of <10%, namely alanyl (membrane) aminopeptidase, also known as CD13. By both multivariate analysis of PLS-DA (Fig. 4) and univariate ANOVA, CD13 is significantly upregulated in the infected rat urine exosome. When the analysis was conducted without gender specificity (Table 4), only CD13 showed an FDR of <10% among these 11 dysregulated proteins. Taking into account gender specificity (Table 2) CD13 was not only significantly upregulated, but also had an FDR of <10%. However, in females, this protein was only significantly upregulated but did not reach the FDR cutoff (Table 3).

**Table 4:** Proteins significantly dysregulated between exosomes from urines of control rats and infected rats.

sl #	gi #	p.value	FDR	% FDR	protein identity
1	149057276	3.76E-06	0.00076	0.0767	alanyl (membrane) aminopeptidase
2	601865	0.010627	0.55605	55.605	aminopeptidase M
3	20806135	0.012682	0.55605	55.605	galectin-3-binding protein precursor
4	133777069	0.01489	0.55605	55.605	Serine (or cysteine) peptidase inhibitor, clade A, member 3K
5	119959830	0.023672	0.55605	55.605	beta-actin
6	57012358	0.037372	0.55605	55.605	keratin, type II cytoskeletal 73
7	392339909	0.038201	0.55605	55.605	PREDICTED: uncharacterized protein LOC679818
8	257467627	0.039191	0.55605	55.605	Fc fragment of IgG binding protein-like precursor
9	149028871	0.041253	0.55605	55.605	annexin A2, isoform CRA_b
10	89573967	0.043033	0.55605	55.605	isocitrate dehydrogenase 1
11	347800746	0.048623	0.55605	55.605	serine protease inhibitor A3K precursor

The VIP scores of proteins identified in the rat urine exosomes shows CD13 to be a top discriminant between infected and uninfected rat urine exosome (Table 1). Twenty-five proteins had a VIP score of >1.5, fulfilling criteria to classify a protein as a reliable discriminant. Our analysis shows that this value 5.72 for CD13.

**Fig 5:**
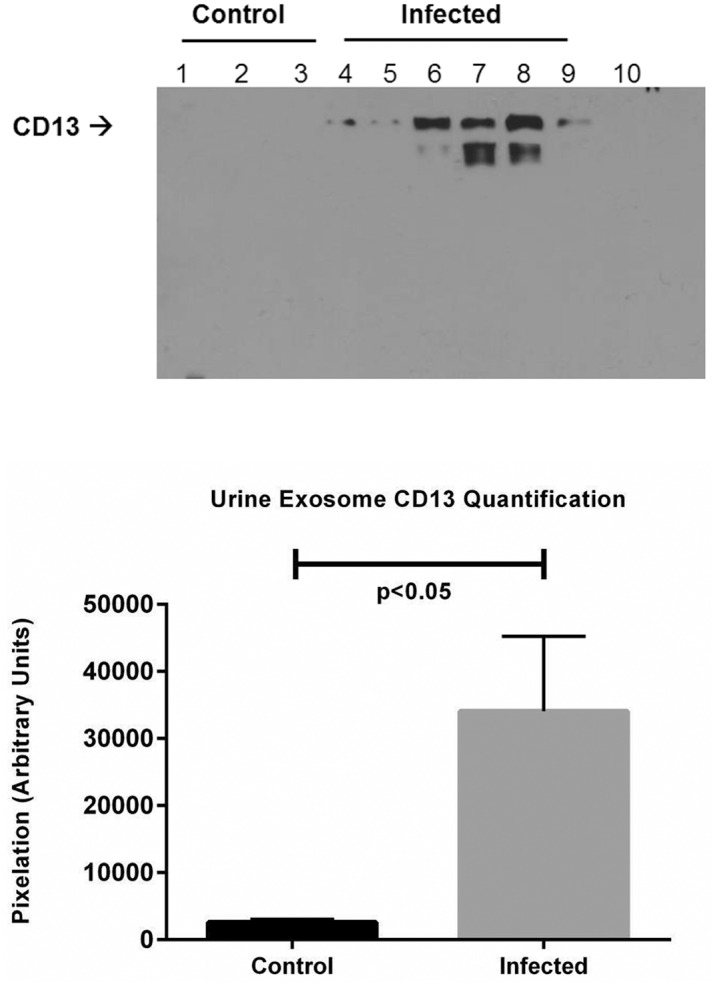
A. Immunoblotting of rat urine exosome for CD13 protein. Lanes 1–3: control; lanes 4–6: lepto infected female; lanes 7–9: infected male. B. Quantification of CD13 from immunoblots in A (control, n = 3; infected, n = 6). Data are means ± SEM. p<0.05 rat urine exosome CD13 *Leptospira*- infected versus uninfected control.

Further, Western immunoblotting of the protein showed that the exosome content of CD13 closely tracked the proteomic data, with a slight increase in the infected female urine exosome, and robust increase in the infected male urine exosome (Fig. 5a). The observed difference was significant (Fig. 5b).

### Tamm-Horsfall Protein is significantly decreased in the infected rat urine

**Fig 6:**
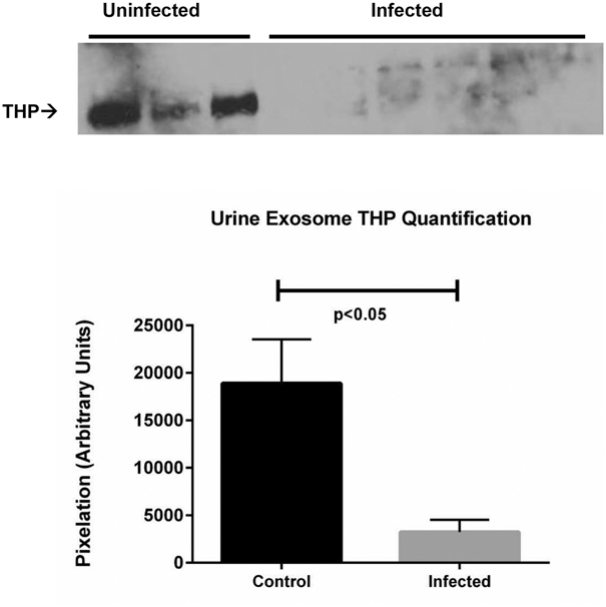
A. Immunoblotting of rat urines for Tamm-Horsfall Protein (THP). Left panel Lanes (1–3): Control Uninfected Rat urines; Right panel Lanes (1–6): *Leptospira*-Infected Rat urines. B. Quantification of THP from immunoblots in A (control, n = 3; infected, n = 6). Data are means ± SEM. p<0.05 rat urine THP, *Leptospira*-infected versus uninfected control.

Given the tubular location of CD13, we tested the hypothesis that other proteins reflecting the tubular function may be affected in response to leptospiral infection. We chose to study the most abundant protein in the normal urine, namely the Tamm-Horsfall Protein (THP). Western immunoblotting of THP in urine of infected and uninfected rats showed robust THP expression in the uninfected rats and significantly lower THP in infected male and female rats, supporting this hypothesis (Fig. 6).

## Discussion

This proteomic analysis of urinary exosomes in a rat leptospiral colonization model identified important biomarkers of infection, including differentially regulated alanine (membrane) aminopeptidase (CD13) and Tamm Horsfall Protein. Further, there were important sex differences of these biomarkers of leptospiral renal tubular colonization, which is particularly provocative given the male predominance of symptomatic and severe leptospirosis universally found in human clinical studies.

In addition, this study has several other important findings. First, to our knowledge this is the first report on urinary exosome protein analysis in an animal model of leptospiral renal colonization. Second, the overwhelming increase in protein expression quality and quantity indicates a specific consequence of leptospiral infection of the organ of leptospiral transmission and provides an important rationale for pursuing such studies in humans that will allow for further understanding of the clinical and biological consequences of chronic renal colonization. Finally, although renal tubular involvement in leptospiral infection has been well-documented, this study is the first to report increased CD13 and decreased Tamm-Horsfall Protein in infection. Although apparently intuitive and in agreement with the published literature in regard to renal tubular involvement in leptospirosis infection, the biological and clinical significance of these findings remains to be determined further.

Leptospirosis is a globally important tropical infectious disease that takes a disproportionate toll in tropical regions [1]. Important gaps remain in translating the know how to reduce the burden of this infectious disease and its pathogenic mechanisms remain poorly understood [4].

Multiple hypothesis-driven studies as well as unbiased approaches have previously focused on unraveling leptospirosis mechanisms [5,6,27,28] [29–31] have considerably advanced our knowledge of the involvement of renal [32–39], pulmonary hemorrhage [40,41] or cardiovascular system components [42] in leptospirosis. However, the host renal functional changes due to *Leptospira* infection are yet to be completely characterized. Given the renal failure rates [29,40,43–48] following leptospirosis, exploring these renal consequences is clinically important. In this study, we found that the infected rat urine shows an overwhelming increase in both quality and quantity of the protein content compared to the uninfected rat urine protein both at the total urine as well as the exosome level.

Importantly, urine exosome CD13 upregulation due to leptospiral infection is robust and consistent, in both male and female rats. Previous work has demonstrated expression of CD13/APN on intestinal and kidney epithelial cell brush border [25,49–52]. CD13 is an ectoenzyme with a multitude of functions: **(a)**. enzymatic cleavage of various peptides such as enkephalins, angiotensins or cytokines [53,54] and their biologic process regulation [55]; **(b)**. marker of differentiation for multiple types of immune cells [56,57] and stem cells [58]; **(c)**. various cellular processes such as proliferation [59] and apoptosis [60], motility [61], chemotaxis [62]; **(d)**. critical role in immunogenic, inflammatory and infection pathways such as antigen processing [63,64] and presentation [65], and phagocytosis in defense against pathogens [66], viral receptor on the host cell surface and the subsequent endocytosis of viruses into cell interior [67–71]. Soluble CD13/APN that circulates in the serum is repeatedly reported to be upregulated at inflammation sites [55,72]. Since CD13 represents a cellular potential for activation or inactivation of inflammatory peptides, modulation of its expression by different agents and stimuli may affect inflammatory and immunologic responses [64,73] and antigen processing [63–65].

Recent evidence [56] shows that CD13 is a multifunctional enzyme with at least 3 different types of activities: (a) peptide cleavage, (b) endocytosis, and (c) signaling. In light of our proteomic data which shows robust and reproducible CD13 upregulation in response to leptospirosis infection, it is possible that either one or all three of these CD13 functionalities are employed. Future studies should further delineate how much or if these functions play a role in mediating specific components of host response to leptospirosis infection. This multifunctional property notwithstanding, so far, CD13/APN activity or its upregulation has not been described specifically in the context of *Leptospira* infection. Nevertheless, relevant to our current findings, a role for the upregulation of APN expression and activity in the membrane is conceivable, and the following features / properties lend this multifunctional enzyme to potentially play a role in *Leptospira* infection/invasion: the viral receptor function of APN [52,69,74] either requires or results in its internalization [55,71]. Previously, the APN/CD13 molecule has been shown to be the predominant component of the detergent-resistant membrane (DRM) microdomains, also called lipid rafts [71]. CD13/APN is shown to play a critical role in appropriate membrane reorganization and protein distribution after a major reshuffling event [75], and is itself is shown to be localized in lipid rafts of the cell membranes [76,77] and endocytosed through physiological sorting mechanisms [57]. At the time of these reports, the concept of exosomes was not as well-established as it is now. The course taken by regular physiologic endocytosis during internalization of specific viruses [69], the role of CD13/APN as the cellular receptor to facilitate this internalization [70] and its critical dependency on the early edosome-formation [71] and subsequent intracellular sub-plasma membrane molecular events bear close resemblance to the formation of exosomes in the renal tubular epithelial cell and their release into urine, the extracellular milieu modeled by the Knepper group [78]. Our proteomic data shows that APN is the most consistently and significantly upregulated protein molecule on the urine exosome of the infected rat. Renal tubular brush border involvement in leptospirosis infection has been well established in animal models showing leptospiral antigen expression in parallel to kidney changes both by light microscopy and electron microscopy examination [79,80]. Further reinforcement of this view comes from the recent evidence that during phagocytosis, CD13 redistribution to phagosome and further internalization increases the efficiency of the process of phagocytosis.

In view of these previous observations and our proteomic data, mechanistically, it is plausible that the host immune system is activated by leptospiral antigens presented to the host immune system. APN located on the plasma membrane of the renal tubular epithelium brush border would be upregulated due to *Leptospira* infection; Though its exact mechanism and its details remain to be elucidated, taken together with the literature our data suggest that APN release from brush border via exosomes in infectious condition, may have been part of the host immune response to leptospiral infection, since it is well-established that exosomes are generated from membranes via endocytic mechanisms [81] and further, that under stress, the exosome cargo changes qualitatively [12,13]. However, if leptospiral infection directly causes increased APN packaging into exosome and its subsequent release needs to be addressed in future. It is plausible that due to the stress of leptospiral infection, renal tubular epithelium mediated exosome packaging is qualitatively altered to include APN. We also found that female rats’ response to *Leptospira* infection is markedly different from that of the male rat. This has important implications and is relevant from the viewpoint of our finding that among asymptomatic human carriers of *Leptospira*, male and female subjects show different phenotypes [10]. However see below, details on the limitations of using or extrapolating the chronic rodent model to an acute infection or a different species such as humans.

The proteomic data also suggested that the renal functions mapping to tubular epithelium may be affected in the *Leptospira* infection. Therefore we tested the hypothesis that the expression of the most abundant protein in the normal healthy urine, Tamm-Horsfall Protein (THP) that is exclusively synthesized in renal tubules should be affected in *Leptospira* infected rats. THP (also called uromodulin) is secreted by the epithelium of thick ascending loop of Henle and the early portion of the distal tubules [82]. One of the phenotypes that a THP knockout mouse model develops is the increased susceptibility to urinary tract infection caused by bacteria [83,84]. THP is a glycosylphosphatidylinositol anchored glycoprotein released from epithelial apical membranes (approximately 120 kDa.) into the tubule fluid by membrane associated phospholipase C [85,86]. Free (urinary) THP has a monomeric weight of approximately 85 kDa with 30% carbohydrate. Our data (Fig. 6) shows that THP expression is reduced in all infected rats. Taken together with the previous work, our data strongly indicate that the tubular epithelial component responsible for THP-synthesis and expression is severely compromised in leptospirosis.

This study design had several limitations. First, the rat model used for our studies is the chronic infection animal model whereas human leptospirosis infection often results in acute infection episodes and often death. The elements of either acute leptospiral infection animal model or human leptospiral infection may yield very different results from those obtained for the model reported here. Even the direction of up/down regulation of significantly different proteins need not necessarily be the same as these chronic animal model findings. However, the methodology that we have employed here namely exosomal analysis, can be eminently applied to study any other model including the acute phases of leptospiral infection in humans or to test the hypothesis that acute infection results in differences in the urine exosome that can be tracked and understood. Secondly, the number of animals analyzed per group is small, reflecting the intensity of this large scale analysis. A higher n value per condition may have increased the robustness of these data further and brought the CD13 differences between uninfected and infected female rats too, to an acceptable FDR of <10%. However this small value of n notwithstanding, these data as such are robust. The difference in protein numbers between infected and uninfected rat urine exosome is remarkably large that it imparted the highest VIP score to CD13. Thirdly, some of the other proteins that feature lower VIP score may have had higher VIP scores by increasing the number of animals. This potentially would have increased our knowledge of what other novel pathways are selected/employed by either leptospires or the host. Finally, we used 1d gel electrophoresis to resolve the exosome proteins prior to LC/MS-MS analysis that resulted in identification of 204 proteins overall. If we had conducted direct exosome protein trypsinization instead of following this method, perhaps the number of identified proteins might have increased. In our experience, exosomes are packaged with non-full length peptides as well, and may have confounded the analysis. By following the gel electrophoresis method, we ensured that we only compare full length protein differences between animals.

In summary, we have characterized host response to leptospiral infection at proteome levels of whole urine and exosomes. Urine is perhaps the most frequently studied biological fluid for exosome proteomics and also one of the most promising biomarker source. Work from the Knepper group shows that many important renal proteins (e.g. aquaporins, polycystins and podocyn) are shed in the urine exosome [87,88]. Our current report adds APN to this group of functionally important renal proteins to be identified in urine exosomes. Our findings suggest that the host response to *Leptospira* infection is gender-specific, and involves renal tubular elements. This report potentially forms the preliminary level basis for our overarching hypothesis that changes in the kidney function as a result of an external insult can be determined using urine exosome analyses.

### Conclusions and future directions

Many conclusions can be drawn from this study:

1. Although the general quantity of proteins in total urine and exosome component show a similar trend, qualitatively the proteins are different based on identifications, the GI numbers and hence the pathways they belong to.
2. Utility of exosome Analysis in characterizing a complex biochemical phenomenon such as the host-response to leptospirosis infection is very high, and has many implications:
  - Public health interest: given that nephron-cell specific cargo that urine exosome carries, primarily the pathway employed or dysregulated for infecting the host to bring about the infection phenotype, and secondly, the response that is unique to each host studied potentially opens up the possibilities of disease-specific and severity-specific treatment to leptospirosis.
  - Non-invasive: it would be ideal to biopsy every infected individual to understand the various facets of leptospirosis infection in order that our knowledge about *Leptospira* increases. However, it is neither practical nor ethical, given the invasive nature. Urine exosome analyses is non-invasive, highly specific and provides a window into the organ level structural or functional changes. If implemented quickly enough in a translational research setting, this specificity potentially imparts ability to move the field towards personalized medicine.
  - Gender-specific host responses to disease onset: our data shows that this methodology of studying differences between male and female host response to infection can be applied in the setting of human disease. Although we should expect different molecules other than CD13 to by dysregulated, the individual-specific pathways mediating different magnitude responses to the same infection can be accurately measured using exosome markers that are either surrogately or directly linked to a particular host-response event. This assumes importance especially in the setting of sub-clinical leptospirosis infection where in the patient does not display any clinical features/symptoms but continues to play host to the bacterium.

## Supporting Information

S1 Text Extended legends and references. (DOCX)

S1 Fig Infected tubule male rat kidney section stain. (TIF)

S2 Fig Infected tubule female rat kidney section stain. (TIF)

S3 Fig Quantitative real time PCR (qPCR) analysis. All urine samples collected were screened for the presence of pathogenic and intermediate-pathogenic Leptospira using a published qPCR TaqMan assay targeting the leptospiral 16S ribosomal gene (1); this assay has been reported in our previous work. Briefly, this was performed using an Opticon 2 real-time PCR machine (MJ Research, USA). The assay protocol was modified from the published version (2) by using the fluorescent probe at a final concentration of 0.2 mM, primers at a final concentration of 0.5 mM, and a 20 mL reaction volume (3). Standard curves for quantification were made using *Leptospira interrogans* serovar Copenhageni strain M20. Standards were prepared as follows. Leptospires were counted using a Petroff-Hauser counting chamber (Hauser Scientific, USA) and serially diluted with sterile double-distilled H2O to 108 to 100 leptospires/ ml. Genomic DNA was subsequently prepared using the DNeasy Tissue Kit (Qiagen, USA). Standards were run in triplicate to generate a standard curve with each run. A negative result was assigned where no amplification occurred before 40 cycles. Controls lacking template were extracted and added to qPCR master mix to detect the presence of contaminating DNA. The raw data is shown in S3 Table.
(TIF)

S4 Fig Clustering with Ward method. the PCA dendrogram cluster with Ward method was performed as summarized in S4 Fig. All female samples cluster together, and all male samples cluster together indicating the difference between the sexes. While the infected females cluster together at one end of the spectrum followed by control female rat exosomes, one of the control males and one of the infected males cluster differently. This may be due to the outbred nature of the rats wherein the level of infection could be different between animals. This condition is reflected in clinical human infection.
(TIF)

S1 Table a: 126 proteins commonly present in urine of uninfected, infected male and infected female rats. b: 156 Proteins Unique to Control rat urine but absent in infected rat urine. c: 272 Proteins unique to infected male rat urine but absent in control animals and infected female rat samples.
(DOCX)

S2 Table a: Proteins commonly present in urinary exosomes uninfected, infected male and infected female rats. b: Proteins unique to control rat urine exosomes, but absent in infected rat urine exosomes. C: Proteins unique to Infected male rat urine exosomes, but absent in control animals and infected female rat samples.
(DOCX)

S3 Table Raw data of *Leptospira* qPCR of rat urine. (DOCX)
