# Correction: Proteomic Analysis of Urine Exosomes Reveals Renal Tubule Response to Leptospiral Colonization in Experimentally Infected Rats

**DOI:** 10.1371/journal.pntd.0003718

**Published:** 2015-04-10

**Authors:** Satish P. RamachandraRao, Michael A. Matthias, Chanthel Kokoy-Mondrogon, Eamon Aghania, Cathleen Park, Casey Kong, Michelle Ishaya, Assael Madrigal, Jennifer Horng, Roni Khoshaba, Anousone Bounkhoun, Fabrizio Basilico, Antonella De Palma, Anna Maria Agresta, Linda Awdishu, Robert K. Naviaux, Joseph M. Vinetz, Pierluigi Mauri

The name of the third author is spelled incorrectly. The correct spelling is: Chanthel Kokoy-Mondrogon. The correct citation is: RamachandraRao SP, Matthias MA, Kokoy-Mondrogon C, Aghania E, Park C, et al. (2015) Proteomic Analysis of Urine Exosomes Reveals Renal Tubule Response to Leptospiral Colonization in Experimentally Infected Rats. PLoS Negl Trop Dis 9(3): e0003640. doi:10.1371/journal.pntd.0003640
