# Correction: Correction: Proteomic Analysis of Urine Exosomes Reveals Renal Tubule Response to Leptospiral Colonization in Experimentally Infected Rats

**DOI:** 10.1371/journal.pntd.0003911

**Published:** 2015-06-24

**Authors:** The PLOS Neglected Tropical Diseases Staff

This correction is to correct a previous correction. The third author’s name is spelled incorrectly. The correct name is Chanthel Kokoy-Mondragon. The correct citation is: RamachandraRao SP, Matthias MA, Kokoy-Mondragon C, Aghania E, Park C, et al. (2015) Proteomic Analysis of Urine Exosomes Reveals Renal Tubule Response to Leptospiral Colonization in Experimentally Infected Rats. PLoS Negl Trop Dis 9(3): e0003640. doi:10.1371/journal.pntd.0003640
