# Supplementary material for: Proteomic Analysis of Urine Exosomes Reveals Renal Tubule Response to Leptospiral Colonization in Experimentally Infected Rats
Source: PLoS Negl Trop Dis. 2015 Mar 20;9(3):e0003640. doi: 10.1371/journal.pntd.0003640 (PMC4368819; doi:10.1371/journal.pntd.0003640)
Supplement: S1 Text — (DOCX) [file pntd.0003640.s001.docx]

**Rat Leptospiruria and kidney infection validation** by Silver Staining Figures S1-S3. *Leptospira* were not detected in urine of Rat #6; while Rat #8 was initially leptospiruric, no *Leptospira* were detected in urine after day 7. In addition, although *Leptospira* was in the urine, the kidney of Rat #6 was positive.

**Figure S1** Infected tubule male rat kidney section stain.

**Figure S2** Infected tubule female rat kidney section stain.

**Figure S3** Quantitative real time PCR (qPCR) Analysis: All urine samples collected were screened for the presence of pathogenic and intermediate-pathogenic Leptospira using a published qPCR TaqMan assay targeting the leptospiral 16S ribosomal gene ([1](#_ENREF_1)); this assay has been reported in our previous work. Briefly, this was performed using an Opticon 2 real-time PCR machine (MJ Research, USA). The assay protocol was modified from the published version ([2](#_ENREF_2)) by using the fluorescent probe at a final concentration of 0.2 mM, primers at a final concentration of 0.5 mM, and a 20 mL reaction volume ([3](#_ENREF_3)). Standard curves for quantification were made using *Leptospira interrogans* serovar Copenhageni strain M20. Standards were prepared as follows. Leptospires were counted using a Petroff-Hauser counting chamber (Hauser Scientific, USA) and serially diluted with sterile double-distilled H2O to 108 to 100 leptospires/ ml. Genomic DNA was subsequently prepared using the DNeasy Tissue Kit (Qiagen, USA). Standards were run in triplicate to generate a standard curve with each run. A negative result was assigned where no amplification occurred before 40 cycles. Controls lacking template were extracted and added to qPCR master mix to detect the presence of contaminating DNA. The raw data is shown in Table S3.

**Figure S4** Clustering with Ward method: the PCA dendrogram cluster with Ward method was performed as summarized in Figure S4. All female samples cluster together, and all male samples cluster together indicating the difference between the sexes. While the infected females cluster together at one end of the spectrum followed by control female rat exosomes, one of the control males and one of the infected males cluster differently. This may be due to the outbred nature of the rats wherein the level of infection could be different between animals. This condition is reflected in clinical human infection.

**References**

1. Smythe LD, Smith IL, Smith GA, Dohnt MF, Symonds ML, Barnett LJ, and McKay DB. A quantitative PCR (TaqMan) assay for pathogenic Leptospira spp. *BMC Infect Dis.* 2002;2(13.

2. Ganoza CA, Matthias MA, Saito M, Cespedes M, Gotuzzo E, and Vinetz JM. Asymptomatic renal colonization of humans in the peruvian Amazon by Leptospira. *PLoS Negl Trop Dis.* 2010;4(2):e612.

3. Ganoza CA, Matthias MA, Collins-Richards D, Brouwer KC, Cunningham CB, Segura ER, Gilman RH, Gotuzzo E, and Vinetz JM. Determining risk for severe leptospirosis by molecular analysis of environmental surface waters for pathogenic Leptospira. *PLoS Med.* 2006;3(8):e308.
