# Supplementary figures and images for: Proteomic Analysis of Urine Exosomes Reveals Renal Tubule Response to Leptospiral Colonization in Experimentally Infected Rats
Source: PLoS Negl Trop Dis. 2015 Mar 20;9(3):e0003640. doi: 10.1371/journal.pntd.0003640 (PMC4368819; doi:10.1371/journal.pntd.0003640)

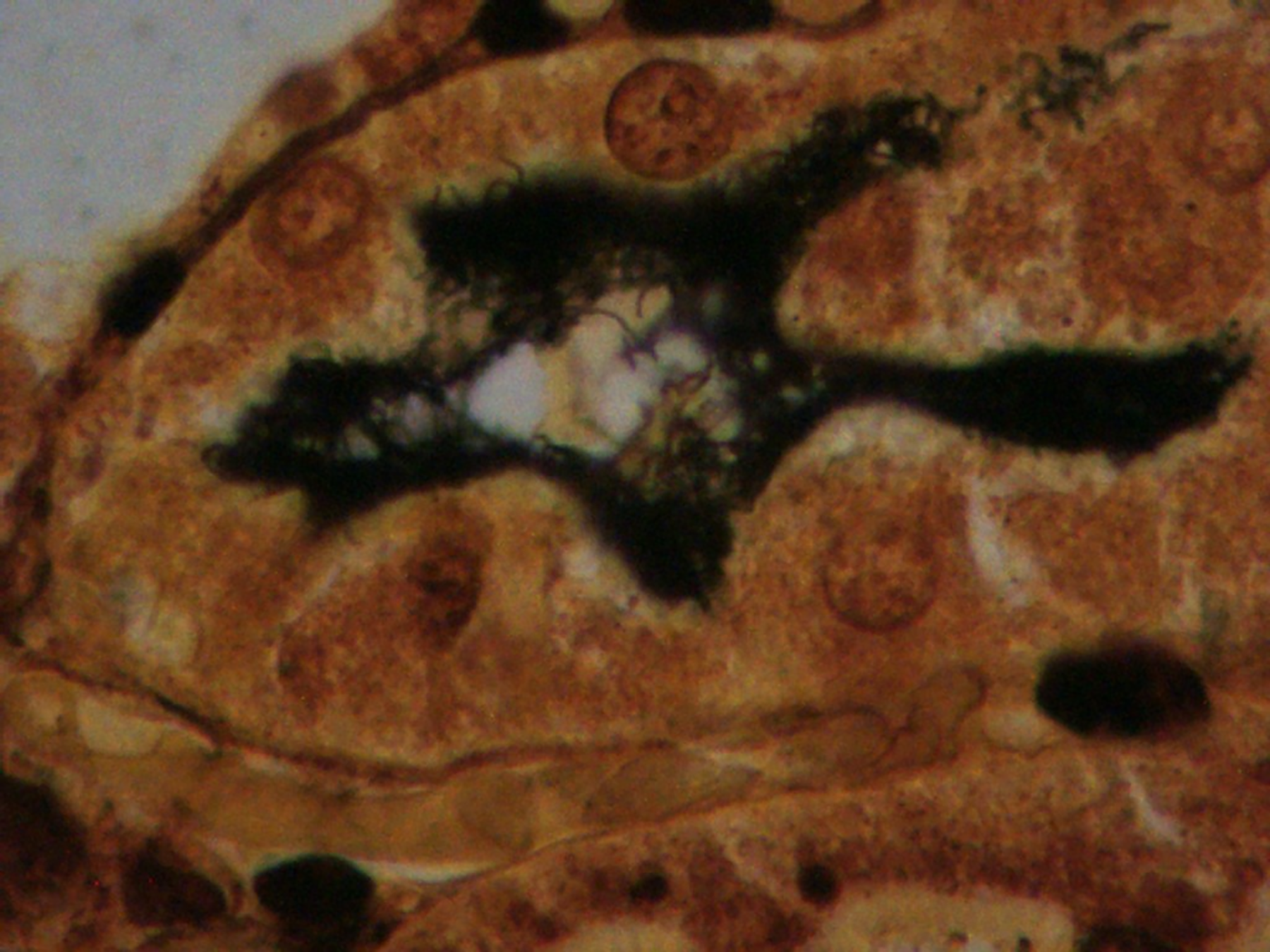

Supplement: S1 Fig — (TIF) [file pntd.0003640.s002.tif]

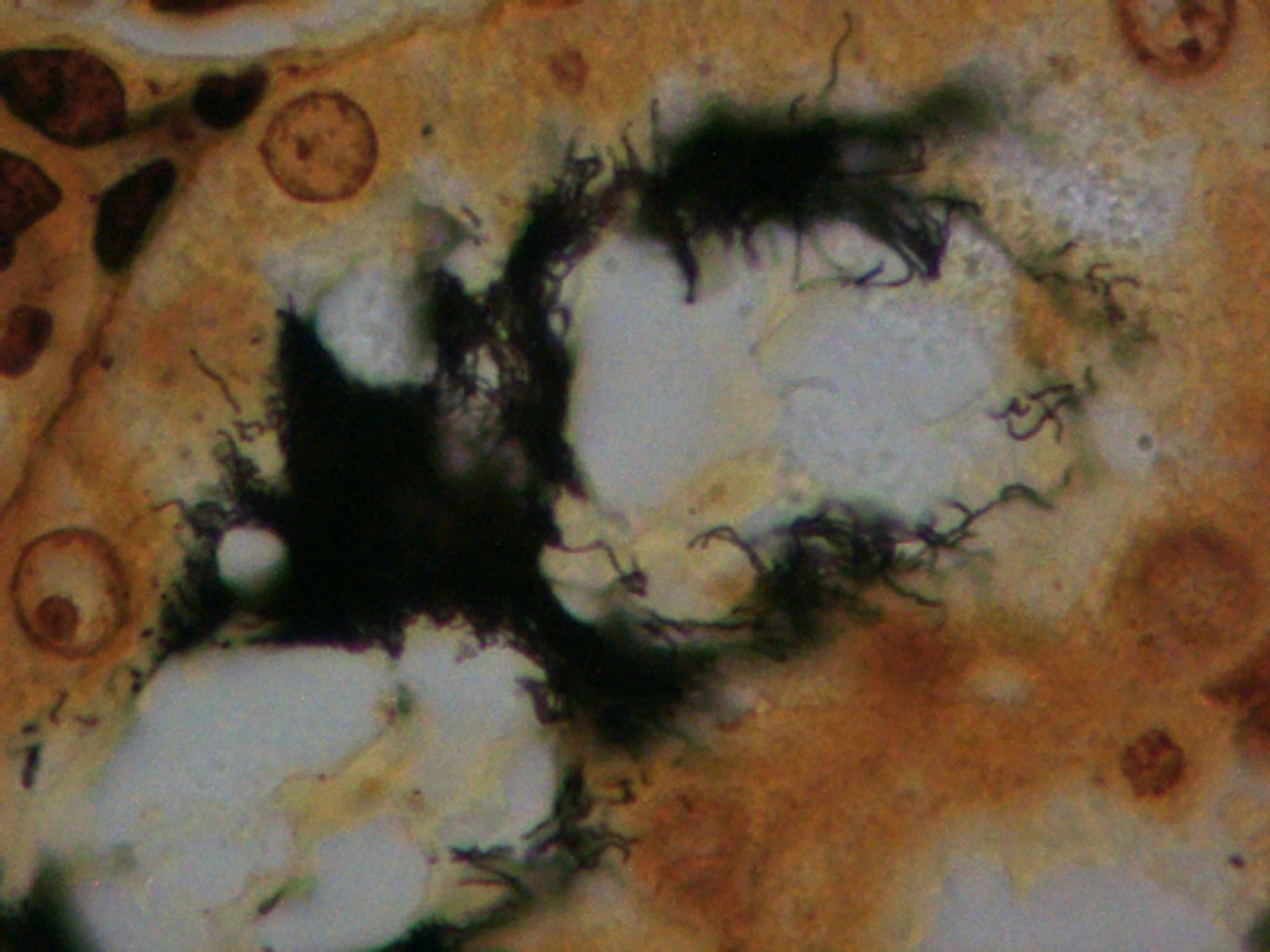

Supplement: S2 Fig — (TIF) [file pntd.0003640.s003.tif]

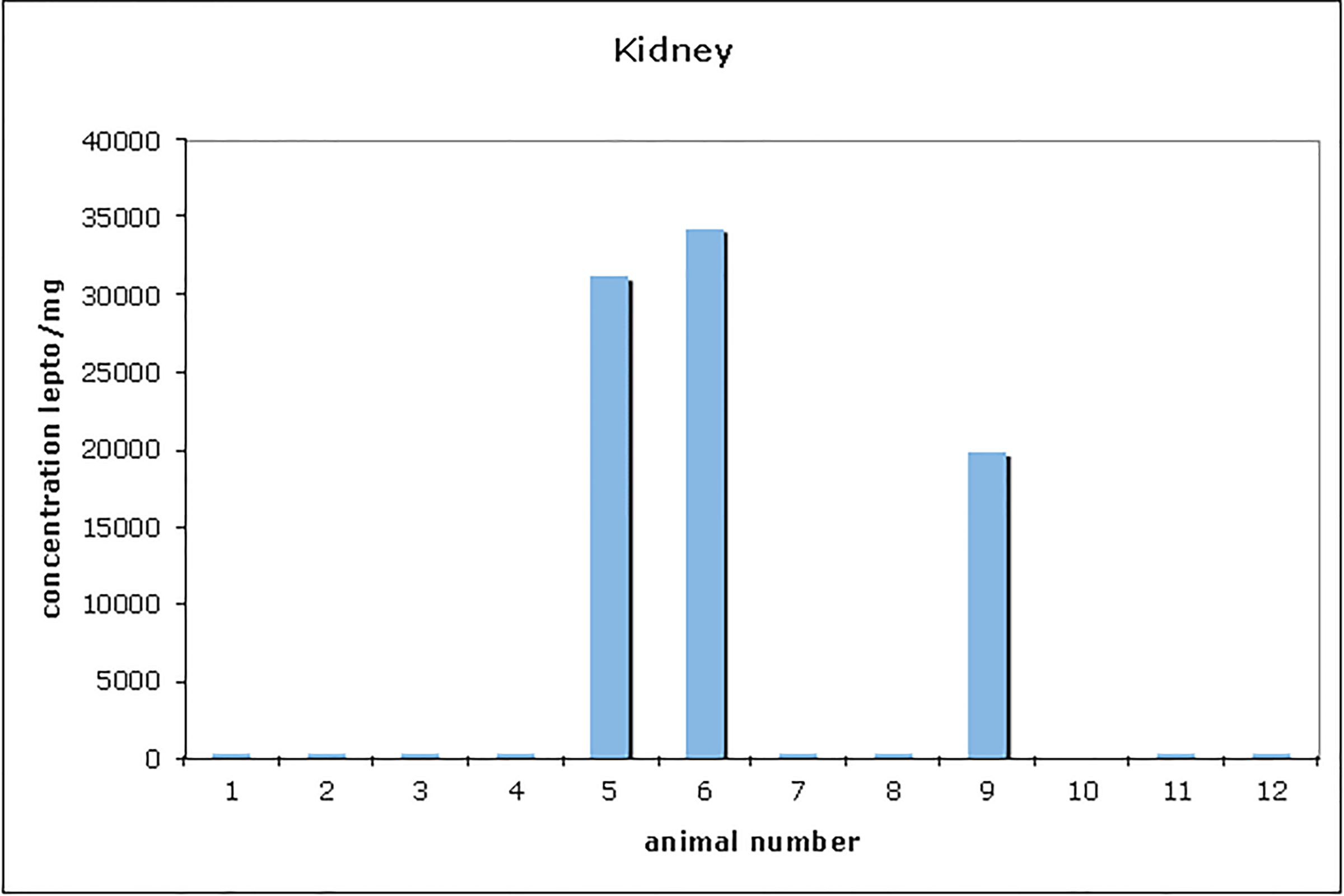

Supplement: S3 Fig — All urine samples collected were screened for the presence of pathogenic and intermediate-pathogenic Leptospira using a published qPCR TaqMan assay targeting the leptospiral 16S ribosomal gene (1); this assay has been reported in our previous work. Briefly, this was performed using an Opticon 2 real-time PCR machine (MJ Research, USA). The assay protocol was modified from the published version (2) by using the fluorescent probe at a final concentration of 0.2 mM, primers at a final concentration of 0.5 mM, and a 20 mL reaction volume (3). Standard curves for quantification were made using Leptospira interrogans serovar Copenhageni strain M20. Standards were prepared as follows. Leptospires were counted using a Petroff-Hauser counting chamber (Hauser Scientific, USA) and serially diluted with sterile double-distilled H2O to 108 to 100 leptospires/ ml. Genomic DNA was subsequently prepared using the DNeasy Tissue Kit (Qiagen, USA). Standards were run in triplicate to generate a standard curve with each run. A negative result was assigned where no amplification occurred before 40 cycles. Controls lacking template were extracted and added to qPCR master mix to detect the presence of contaminating DNA. The raw data is shown in S3 Table. (TIF) [file pntd.0003640.s004.tif]

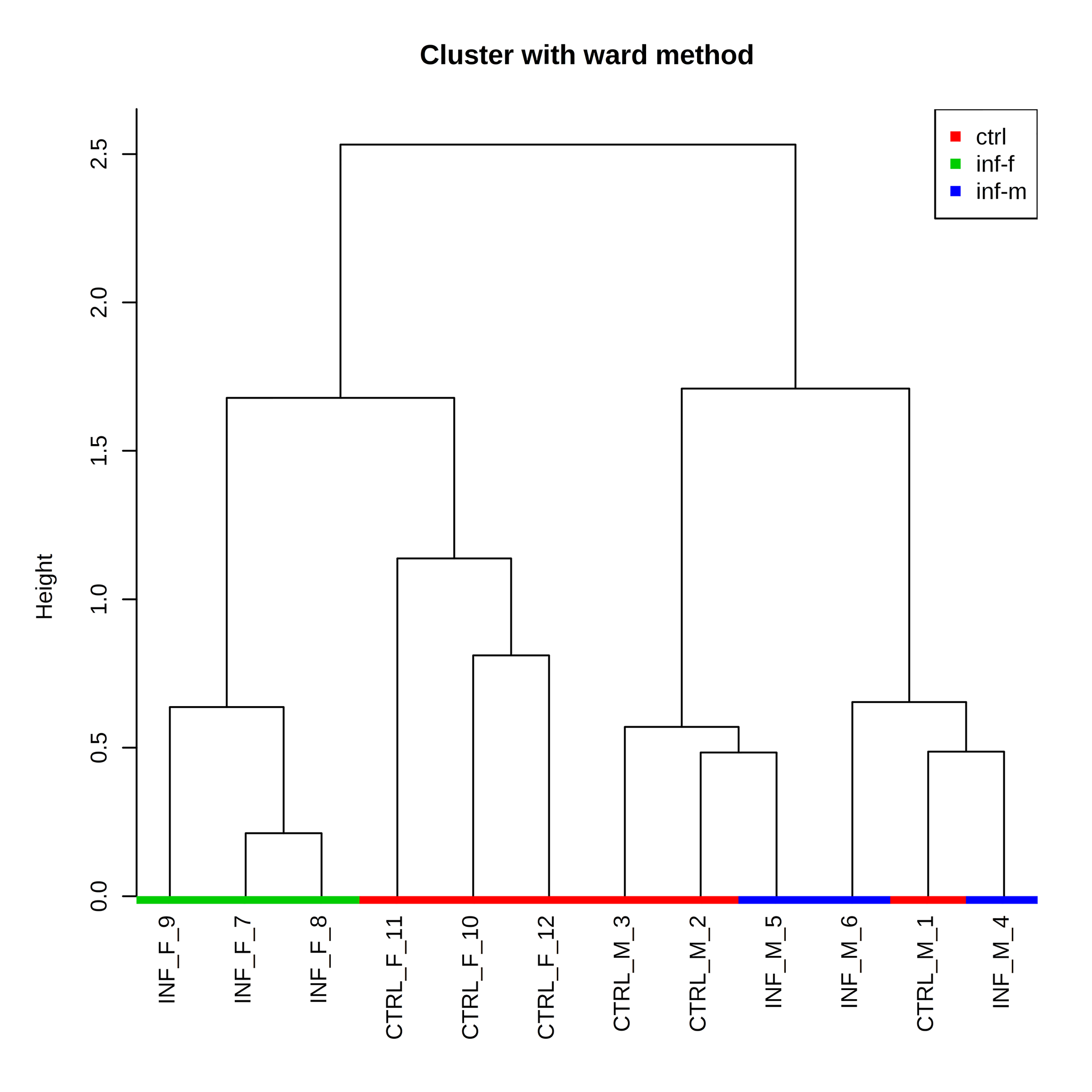

Supplement: S4 Fig — the PCA dendrogram cluster with Ward method was performed as summarized in S4 Fig. All female samples cluster together, and all male samples cluster together indicating the difference between the sexes. While the infected females cluster together at one end of the spectrum followed by control female rat exosomes, one of the control males and one of the infected males cluster differently. This may be due to the outbred nature of the rats wherein the level of infection could be different between animals. This condition is reflected in clinical human infection. (TIF) [file pntd.0003640.s005.tif]
