# Supplementary material for: Proteomic Analysis of Urine Exosomes Reveals Renal Tubule Response to Leptospiral Colonization in Experimentally Infected Rats
Source: PLoS Negl Trop Dis. 2015 Mar 20;9(3):e0003640. doi: 10.1371/journal.pntd.0003640 (PMC4368819; doi:10.1371/journal.pntd.0003640)
Supplement: S1 Table — b: 156 Proteins Unique to Control rat urine but absent in infected rat urine. c: 272 Proteins unique to infected male rat urine but absent in control animals and infected female rat samples. (DOCX) [file pntd.0003640.s006.docx]

| **Table S1-a. 126 Proteins commonly present in urine of all groups:**  **Uninfected, infected male and infected female rats** | | |
| --- | --- | --- |
| **Sl #** | **Gene ID** | **Name of the Protein** |
| 1 | 57232736 | 11 kDa protein 2 |
| 2 | 157823471 | 6-phosphogluconolactonase |
| 3 | 81872093 | A1M_RAT Alpha-1-macroglobulin precursor (Alpha-1-M) |
| 4 | 117647198 | Adipsin |
| 5 | 158138568 | albumin |
| 6 | 149064290 | aldehyde dehydrogenase family 7, member A1, isoform |
| 7 | 6978497 | alpha 1 microglobulin/bikunin |
| 8 | 83816939 | alpha-1-inhibitor III |
| 9 | 1196815 | alpha-2u globulin |
| 10 | 8307696 | alpha-2u globulin |
| 11 | 22219450 | alpha-2u globulin PGCL1 |
| 12 | 54020747 | alpha-2u globulin PGCL2 |
| 13 | 22219456 | alpha-2u globulin PGCL5 |
| 14 | 58293772 | amylase 1, salivary |
| 15 | 13928684 | amylase 2, pancreatic |
| 16 | 6978505 | annexin A5 |
| 17 | 1929920 | anti-idiotype immunoglobulin M light chain |
| 18 | 4096754 | anti-NGF30 antibody light-chain |
| 19 | 7549746 | beta-2-microglobulin |
| 20 | 8393197 | C-reactive protein, pentraxin-related |
| 21 | 13786164 | cadherin 1 |
| 22 | 58865686 | cadherin 16 |
| 23 | 14010887 | calbindin 1 |
| 24 | 4433355 | carboxyesterase E1 |
| 25 | 1705630 | CATB_RAT Cathepsin B precursor (Cathepsin B1) (RSG-2) |
| 26 | 58865488 | cathepsin A |
| 27 | 20806147 | CD48 antigen |
| 28 | 27545443 | CEA-related cell adhesion molecule 10 |
| 29 | 461756 | CLUS_RAT Clusterin precursor (Sulfated glycoprotein 2) (SGP- |
| 30 | 116597 | CO3_RAT Complement C3 precursor |
| 31 | 4090263 | collagen alpha 1 type X |
| 32 | 82830409 | colonic system B0+ amino acid transporter |
| 33 | 19424346 | common salivary protein 1 |
| 34 | 220698 | contrapsin-like protease inhibitor (CPi-21) |
| 35 | 1213217 | Cu/Zn superoxide dismutase |
| 36 | 54234046 | cystatin C |
| 37 | 6978769 | deoxyribonuclease I |
| 38 | 76096314 | desmocollin 2 |
| 39 | 6978773 | dipeptidylpeptidase 4 |
| 40 | 14010871 | dipeptidylpeptidase 7 |
| 41 | 38303863 | Egf protein |
| 42 | 6978797 | epidermal growth factor |
| 43 | 31745156 | F-box protein 11 |
| 44 | 17865327 | fetuin beta |
| 45 | 17865331 | fibroblast growth factor receptor 3 |
| 46 | 120178 | FINC_RAT Fibronectin precursor (FN) |
| 47 | 6978890 | gamma-glutamyl hydrolase |
| 48 | 51854227 | gelsolin |
| 49 | 51591907 | glandular kallikrein 11 |
| 50 | 6980960 | glycosylation dependent cell adhesion molecule 1 |
| 51 | 6978879 | group specific component |
| 52 | 2851391 | HA12_RAT RT1 class I histocompatibility antigen, AA alpha c |
| 53 | 16758014 | hemopexin |
| 54 | 46485726 | hyaluronoglucosaminidase 1 |
| 55 | 66730268 | hypothetical protein LOC500124 |
| 56 | 7650274 | immunoglobulin kappa light chain variable region |
| 57 | 7650286 | immunoglobulin kappa light chain variable region |
| 58 | 7650290 | immunoglobulin kappa light chain variable region |
| 59 | 7650294 | immunoglobulin kappa light chain variable region |
| 60 | 7650298 | immunoglobulin kappa light chain variable region |
| 61 | 89258021 | immunoglobulin lambda light chain |
| 62 | 204698 | immunoglobulin light chain |
| 63 | 61556795 | insulin-like growth factor binding protein 7 |
| 64 | 158534071 | interleukin 4 receptor |
| 65 | 149044029 | jagged 2, isoform CRA_a |
| 66 | 125144 | KACB_RAT Ig kappa chain C region, B allele |
| 67 | 818030 | kallikrein |
| 68 | 61556850 | kallikrein 6 |
| 69 | 6981132 | kallikrein 7 |
| 70 | 13591914 | kidney aminopeptidase M |
| 71 | 169234844 | lectin, mannose-binding 2 |
| 72 | 56789710 | LOC500180 protein |
| 73 | 68534280 | LOC500183 protein |
| 74 | 13562118 | low density lipoprotein receptor-related protein 2 |
| 75 | 758263 | major acute phase alpha-1 |
| 76 | 38454302 | major urinary protein 4 |
| 77 | 55742881 | matrix-remodelling associated 8 |
| 78 | 6981196 | meprin 1 alpha |
| 79 | 13928928 | napsin A aspartic peptidase |
| 80 | 26023947 | neuropilin 1 |
| 81 | 161760644 | nucleolin |
| 82 | 16757980 | orosomucoid 1 |
| 83 | 157818431 | periplakin |
| 84 | 123779278 | PGAP2_RAT Post-GPI attachment to proteins factor 2 (FGF |
| 85 | 56605734 | poly (ADP-ribose) polymerase family, member 3 |
| 86 | 27151742 | polymeric immunoglobulin receptor |
| 87 | 109474941 | PREDICTED: similar to alpha2u globulin |
| 88 | 62647341 | PREDICTED: similar to CG5815-PA, isoform A |
| 89 | 109458674 | PREDICTED: similar to Glandular kallikrein-10 prec |
| 90 | 109509284 | PREDICTED: similar to ICOS ligand precursor (B7 ho |
| 91 | 109475037 | PREDICTED: similar to Receptor-type tyrosine-prote |
| 92 | 109469079 | PREDICTED: similar to Vomeromodulin |
| 93 | 149037632 | procollagen, type VI, alpha 3 (predicted), isoform C |
| 94 | 12083677 | prolactin induced protein |
| 95 | 6981430 | prostaglandin H2 D-isomerase |
| 96 | 6981420 | protease, serine, 1 (trypsin 1) |
| 97 | 149019823 | rCG36811 |
| 98 | 149026570 | rCG37802 |
| 99 | 149032334 | rCG39036, isoform CRA_a |
| 100 | 149062636 | rCG48145 |
| 101 | 149037297 | rCG53373 |
| 102 | 149036353 | rCG56561 |
| 103 | 149027662 | rCG64160 |
| 104 | 149037009 | rCG64255 |
| 105 | 149037292 | rCG64257 |
| 106 | 149037307 | rCG64263 |
| 107 | 149047040 | rCG64315 |
| 108 | 92401 | S06084 Ig kappa chain precursor |
| 109 | 92404 | S12954 Ig kappa chain V region (Y13-259) |
| 110 | 479224 | S32806 Ig kappa chain precursor - rat (fragment) |
| 111 | 57527135 | serine (or cysteine) peptidase inhibitor, clade |
| 112 | 6981576 | serine protease inhibitor 2b |
| 113 | 51036655 | serine protease inhibitor alpha 1 |
| 114 | 148747488 | serum amyloid P-component |
| 115 | 149043610 | similar to B7-like protein GL50-B (predicted), isofo |
| 116 | 149046165 | similar to hypothetical protein DKFZp434D2328 (predi |
| 117 | 2507387 | SPA3L_RAT Serine protease inhibitor A3L precursor (Serpin A |
| 118 | 2507388 | SPA3N_RAT Serine protease inhibitor A3N precursor (Serpin A |
| 119 | 38512111 | Tpi1 protein |
| 120 | 1854476 | transferrin |
| 121 | 31560030 | tropomyosin 1, alpha |
| 122 | 136467 | TTHY_RAT Transthyretin precursor (Prealbumin) (TBPA) |
| 123 | 149026097 | unc-5 homolog C (C. elegans) |
| 124 | 55628 | unnamed protein product |
| 125 | 220649 | unnamed protein product |
| 126 | 8394509 | uromodulin |

| **Table S1-b: 156 Proteins Unique to Control rat urine: Absent in infected rat urine** | | |
| --- | --- | --- |
| **SI #** | **Gene Id** | **Name of the protein** |
| 1 | 66730294 | abhydrolase domain containing 12 |
| 2 | 149059068 | adaptor-related protein complex 3, beta 1 subunit (p |
| 3 | 77020246 | adenylate cyclase 6 |
| 4 | 5163368 | AF145445_1 chromogranin A precursor |
| 5 | 4096752 | anti-NGF30 antibody heavy-chain |
| 6 | 71043734 | asparaginyl-tRNA synthetase |
| 7 | 16758088 | ATX1 (antioxidant protein 1) homolog 1 |
| 8 | 51854219 | bactericidal/permeability-increasing protein |
| 9 | 157821429 | bromodomain adjacent to zinc finger domain, 2A |
| 10 | 149025901 | calcium/calmodulin-dependent protein kinase II, delt |
| 11 | 16758414 | carboxypeptidase B2 (plasma) |
| 12 | 58865900 | CD97 antigen |
| 13 | 81883636 | CL062_RAT Uncharacterized protein C12orf62 homolog |
| 14 | 187282052 | coiled-coil domain containing 114 |
| 15 | 13162353 | complement factor I |
| 16 | 114145489 | deltex 4 homolog |
| 17 | 6978585 | discoidin domain receptor family, member 1 |
| 18 | 157822779 | DnaJ (Hsp40) homolog, subfamily C, member 11 |
| 19 | 58865748 | dual specificity phosphatase 2 |
| 20 | 157823277 | dysferlin |
| 21 | 114051321 | eukaryotic translation initiation factor 4A, isoform 3 |
| 22 | 157822213 | Fez family zinc finger 2 |
| 23 | 6680007 | gap junction protein, gamma 1 |
| 24 | 29789269 | glutamate receptor, ionotropic, AMPA1 (alpha 1) |
| 25 | 6980984 | glutamate receptor, ionotropic, N-methyl D-aspartate 2B |
| 26 | 8393519 | H2A histone family, member Y |
| 27 | 34734058 | hemopoietic cell kinase |
| 28 | 6978785 | heparin-binding EGF-like growth factor |
| 29 | 2642598 | high molecular-weight neurofilament |
| 30 | 158711670 | homeo box A7 |
| 31 | 149036004 | homer homolog 3 (Drosophila), isoform CRA_c |
| 32 | 157817891 | hypothetical protein LOC296188 |
| 33 | 157823001 | hypothetical protein LOC299356 |
| 34 | 157821563 | hypothetical protein LOC303070 |
| 35 | 157821469 | hypothetical protein LOC305142 |
| 36 | 114145551 | hypothetical protein LOC360845 |
| 37 | 88853861 | hypothetical protein LOC362319 |
| 38 | 66730469 | hypothetical protein LOC500392 |
| 39 | 187282311 | hypothetical protein LOC686539 |
| 40 | 149059900 | hypothetical protein RDA279, isoform CRA_c |
| 41 | 7650280 | immunoglobulin kappa light chain variable region |
| 42 | 40018574 | inhibitor of kappaB kinase gamma |
| 43 | 157819773 | interleukin 20 receptor, alpha |
| 44 | 6981112 | isovaleryl Coenzyme A dehydrogenase |
| 45 | 157820161 | junctophilin 1 |
| 46 | 8393820 | L1 cell adhesion molecule |
| 47 | 157822983 | La ribonucleoprotein domain family, member 6 |
| 48 | 149060659 | leishmanolysin-like (metallopeptidase M8 family) (predicted), isoform CRA_b |
| 49 | 157821311 | leucine rich repeat containing 19 |
| 50 | 50845391 | lymphocyte antigen 6 complex G5B |
| 51 | 157820271 | lysyl oxidase-like 4 |
| 52 | 149043694 | minichromosome maintenance deficient 3 (S. cerevisiae) associated protein (predicted), isoform CRA_a |
| 53 | 71043904 | mitochondrial translational release factor 1-like |
| 54 | 13027473 | MRS2 magnesium homeostasis factor homolog |
| 55 | 157819037 | muscleblind-like 3 |
| 56 | 11177868 | myc-like oncogene, s-myc protein |
| 57 | 53850594 | NEDD4 binding protein 2-like 2 |
| 58 | 62903398 | neuregulin-1 beta1b |
| 59 | 157820741 | NIMA (never in mitosis gene a)-related expressed kinase 7 |
| 60 | 157817338 | olfactomedin-like 2A |
| 61 | 47576035 | olfactory receptor Olr1436 |
| 62 | 47575915 | olfactory receptor Olr1468 |
| 63 | 47576369 | olfactory receptor Olr1549 |
| 64 | 47576923 | olfactory receptor Olr23 |
| 65 | 47577673 | olfactory receptor Olr563 |
| 66 | 47578083 | olfactory receptor Olr624 |
| 67 | 157820277 | oligodendrocyte transcription factor 3 |
| 68 | 11968064 | parvalbumin |
| 69 | 56839 | peptidylglycine alpha-amidating monooxygenase |
| 70 | 109463879 | PREDICTED: hypothetical protein |
| 71 | 109468519 | PREDICTED: hypothetical protein |
| 72 | 109507934 | PREDICTED: hypothetical protein |
| 73 | 109511480 | PREDICTED: hypothetical protein |
| 74 | 109510313 | PREDICTED: similar to adenylate kinase 1 |
| 75 | 109464904 | PREDICTED: similar to arylacetamide deacetylase |
| 76 | 109507439 | PREDICTED: similar to CG8486-PA, isoform A |
| 77 | 109509319 | PREDICTED: similar to Collagen alpha-1(XVIII) chain precursor |
| 78 | 62643838 | PREDICTED: similar to Cornifin A (Small proline-rich protein 1A) (SPR1A) (SPRR1) |
| 79 | 109509251 | PREDICTED: similar to dynein, axonemal, heavy chain 8 |
| 80 | 109458635 | PREDICTED: similar to F49E2.5d |
| 81 | 109510977 | PREDICTED: similar to Fibronectin type-III domain-containing protein 3a |
| 82 | 109470173 | PREDICTED: similar to FLJ44048 protein |
| 83 | 109511263 | PREDICTED: similar to G-protein coupled receptor 112 |
| 84 | 109470509 | PREDICTED: similar to Gamma-tubulin complex component 4 (GCP-4) |
| 85 | 109506294 | PREDICTED: similar to GREB1 protein isoform a |
| 86 | 109470956 | PREDICTED: similar to GTPase activating Rap/RanGAP domain-like 1 isoform 1 |
| 87 | 109488972 | PREDICTED: similar to keratin associated protei |
| 88 | 109460042 | PREDICTED: similar to kinesin family member 11 |
| 89 | 109478802 | PREDICTED: similar to leucine zipper protein 5 |
| 90 | 109498276 | PREDICTED: similar to lin-9 homolog |
| 91 | 62658603 | PREDICTED: similar to paired immunoglobin-like type |
| 92 | 109481099 | PREDICTED: similar to patatin-like phospholipase domain containing 5 |
| 93 | 109458215 | PREDICTED: similar to phospholipase A2, group I |
| 94 | 109514763 | PREDICTED: similar to Polycomb group RING finger protein 2 (DNA-binding protein Mel-18) (RING finger protein 110) (Zinc finger protein 144) (Zfp- |
| 95 | 109475427 | PREDICTED: similar to potassium voltage-gated channel KQT-like protein 4 isoform a |
| 96 | 109506521 | PREDICTED: similar to protocadherin 1 isoform 2 precursor |
| 97 | 109475770 | PREDICTED: similar to regulatory solute carrier protein, family 1, member 1 |
| 98 | 109468922 | PREDICTED: similar to Ribosome-binding protein 1 (Ribosome receptor protein) (mRRp) |
| 99 | 109458945 | PREDICTED: similar to RIKEN cDNA 5730590G19-like |
| 100 | 109499392 | PREDICTED: similar to RuvB-like 2 |
| 101 | 109504697 | PREDICTED: similar to semaphorin 4D |
| 102 | 109485951 | PREDICTED: similar to Semaphorin-4C precursor (Semaphorin I) (Sema I) (Semaphorin C-like 1) (M-Sema F) |
| 103 | 109497755 | PREDICTED: similar to serine (or cysteine) prot |
| 104 | 62666648 | PREDICTED: similar to shroom |
| 105 | 109496642 | PREDICTED: similar to sidekick homolog 1 |
| 106 | 62656181 | PREDICTED: similar to Smith-Magenis syndrome chromosome region, candidate 8 homolog |
| 107 | 109513245 | PREDICTED: similar to spermatogenesis associated glutamate (E)-rich protein 4d |
| 108 | 109502809 | PREDICTED: similar to TBC1 domain family member 4 (Akt substrate of 160 kDa) (AS160) isoform 3 |
| 109 | 109509498 | PREDICTED: similar to Temporarily Assigned Gene name family member (tag-278) |
| 110 | 109492050 | PREDICTED: similar to Temporarily Assigned Gene name family member (tag-58) |
| 111 | 109470142 | PREDICTED: similar to titin isoform N2-B |
| 112 | 109486867 | PREDICTED: similar to translocating chain-associating membrane protein 2 |
| 113 | 109460116 | PREDICTED: similar to Transmembrane 9 superfamily protein member 3 precursor |
| 114 | 109486727 | PREDICTED: similar to transmembrane protein 63b isoform 2 |
| 115 | 109464629 | PREDICTED: similar to zinc finger homeodomain 4 |
| 116 | 109510913 | PREDICTED: similar to Zinc finger MYM-type protein 3 (Zinc finger protein 261) (DXHXS6673E protein) |
| 117 | 109468626 | PREDICTED: similar to zinc finger protein 239 (predicted) |
| 118 | 109483463 | PREDICTED: similar to zinc finger protein 291 |
| 119 | 62644851 | PREDICTED: similar to zyg-11 homolog B (C. elegans)-like isoform 2 |
| 120 | 157786586 | protein kinase, membrane associated tyrosine/threonine 1 |
| 121 | 157821043 | protein phosphatase 1, regulatory (inhibitor) subunit 12B |
| 122 | 149046615 | putative homeodomain transcription factor 2 (predicted), isoform CRA_b |
| 123 | 149039761 | rCG24253 |
| 124 | 149018564 | rCG25804, isoform CRA_e |
| 125 | 149022521 | rCG27063 |
| 126 | 149037091 | rCG31965 |
| 127 | 149042912 | rCG32401, isoform CRA_a |
| 128 | 149053375 | rCG34104, isoform CRA_a |
| 129 | 149019791 | rCG36779 |
| 130 | 149031261 | rCG41929, isoform CRA_a |
| 131 | 149057799 | rCG42974 |
| 132 | 149045167 | rCG43947 |
| 133 | 149030350 | rCG52111 |
| 134 | 149061377 | rCG63640 |
| 135 | 4506661 | ribosomal protein L7a |
| 136 | 48428500 | S40A1_RAT Solute carrier family 40 member 1 (Ferroportin-1) (Cell adhesion regulator) (CAR1) |
| 137 | 9507139 | salivary protein 1 |
| 138 | 157823305 | SET and MYND domain containing 5 |
| 139 | 157824020 | SET domain containing 2 |
| 140 | 57527612 | sialin |
| 141 | 149027597 | similar to endoplasmic oxidoreductase 1 beta (predic |
| 142 | 149056582 | similar to hypothetical protein FLJ10241, isoform CR |
| 143 | 149035478 | similar to hypothetical protein MGC17299 (predicted), isoform CRA_a |
| 144 | 157818625 | solute carrier family 2 (facilitated glucose transporter), member 12 |
| 145 | 149022685 | solute carrier family 35, member C1 (predicted), isoform CRA_b |
| 146 | 1236523 | SRC-CD44s |
| 147 | 158711736 | structural maintenance of chromosomes 2-like 1 |
| 148 | 62079097 | T-cell immunoglobulin and mucin domain containing 2 |
| 149 | 183013175 | tenascin C |
| 150 | 157817646 | TraB domain containing |
| 151 | 51591911 | transmembrane protease, serine 11b |
| 152 | 127139541 | tripartite motif-containing 21 |
| 153 | 46485190 | ubiquitin conjugation factor E4 A |
| 154 | 169642255 | Unknown (protein for MGC:188246) |
| 155 | 76096338 | zinc finger E-box binding homeobox 2 |
| 156 | 58865498 | zinc finger, SWIM domain containing 2 |

| **Table S1-c: 272 Proteins unique to Infected male rat urine:**  **absent in control animals and infected female rat samples** | | |
| --- | --- | --- |
| **Sl #** | **Gene ID** | **Name of the Protein** |
| 1 | 33086498 | Aa2-001 |
| 2 | 61557012 | ABI gene family, member 3 |
| 3 | 11559962 | acetyl-coenzyme A carboxylase alpha |
| 4 | 157821879 | activating transcription factor 6 |
| 5 | 157821693 | acute myelogenous leukemia 1 translocation 1 pr |
| 6 | 21311256 | AF401635_1 Rho GTPase activating protein 4 |
| 7 | 20271387 | AF436847_1 complement factor H-related protein |
| 8 | 27229290 | afamin |
| 9 | 71051720 | Akap9 protein |
| 10 | 31377518 | alkaline phosphatase 1, intestinal, defined by SSR |
| 11 | 33302595 | AMPE_RAT Glutamyl aminopeptidase (EAP) (Aminopeptidase A) |
| 12 | 155369656 | aquarius |
| 13 | 114145538 | arylsulfatase J |
| 14 | 17365957 | ATRX_RAT Transcriptional regulator ATRX (ATP-dependent hel |
| 15 | 61557097 | Axl receptor tyrosine kinase isoform 2 |
| 16 | 157818469 | bactericidal/permeability-increasing protein-li |
| 17 | 13929056 | basal cell adhesion molecule |
| 18 | 157816957 | BTB (POZ) domain containing 7 |
| 19 | 20302073 | cadherin 13 |
| 20 | 157823195 | cartilage intermediate layer protein, nucleotid |
| 21 | 8393221 | cathepsin S preproprotein |
| 22 | 115743 | CATL1_RAT Cathepsin L1 precursor (Major excreted protein) |
| 23 | 13027412 | caudal type homeo box 2 |
| 24 | 543960 | CBS_RAT Cystathionine beta-synthase (Serine sulfhydrase) (Be |
| 25 | 13591932 | chondroitin sulfate proteoglycan 4 |
| 26 | 9506949 | contactin 3 |
| 27 | 157818697 | coronin, actin binding protein 1C |
| 28 | 68052366 | CP033_MOUSE U11/U12 small nuclear ribonucleoprotein 25 k |
| 29 | 56013 | CRP2 |
| 30 | 12408314 | cysteine-rich secretory protein 1 |
| 31 | 149068218 | cytochrome P450, family 2, subfamily r, polypeptide |
| 32 | 62079155 | DALR anticodon binding domain containing 3 |
| 33 | 62078923 | DAZ interacting protein 1-like |
| 34 | 157818387 | DEAH (Asp-Glu-Ala-His) box polypeptide 36 |
| 35 | 6683021 | decay accelerating factor (GPI-form) |
| 36 | 258144 | dopamine D1 receptor; D1 receptor |
| 37 | 149027910 | dpy-19-like 1 (C. elegans) (predicted), isoform CRA_ |
| 38 | 157819323 | dual adaptor for phosphotyrosine and 3-phosphoi |
| 39 | 13928768 | esterase 22 |
| 40 | 9506993 | eukaryotic translation initiation factor 2-alpha kin |
| 41 | 56268935 | Fbxl13 protein |
| 42 | 126722908 | Fbxw17 protein |
| 43 | 47059189 | G7c protein |
| 44 | 157818237 | galactose-3-O-sulfotransferase 4 |
| 45 | 149055434 | galactosidase, alpha (mapped), isoform CRA_a |
| 46 | 55741776 | gamma sarcoglycan |
| 47 | 145280505 | gamma-glutamyltransferase 1 |
| 48 | 13027414 | glyceraldehyde-3-phosphate dehydrogenase, spermatog |
| 49 | 47059179 | heat shock 70kD protein 1B |
| 50 | 12750773 | heparin-binding fibroblast growth factor receptor 2 |
| 51 | 157819447 | hypothetical protein LOC300723 |
| 52 | 40786481 | hypothetical protein LOC304055 |
| 53 | 157818553 | hypothetical protein LOC308004 |
| 54 | 62078781 | hypothetical protein LOC309029 |
| 55 | 155369696 | hypothetical protein LOC683313 |
| 56 | 56789486 | Igh-1a protein |
| 57 | 23559227 | immunoglobulin alpha heavy chain |
| 58 | 62078619 | Immunoglobulin heavy chain (gamma polypeptide) |
| 59 | 6900850 | immunoglobulin heavy chain variable region |
| 60 | 89258019 | immunoglobulin lambda light chain |
| 61 | 89258025 | immunoglobulin lambda light chain |
| 62 | 89258029 | immunoglobulin lambda light chain |
| 63 | 28461159 | kallikrein, submaxillary gland S3 |
| 64 | 8393610 | karyopherin (importin) beta 1 |
| 65 | 149051019 | kinase D-interacting substance 220, isoform CRA_a |
| 66 | 157817668 | L-amino acid oxidase 1 |
| 67 | 157786856 | lactoperoxidase |
| 68 | 6981154 | lectin, galactose binding, soluble 5 |
| 69 | 2851467 | LEG9_RAT Galectin-9 (36 kDa beta-galactoside-binding lectin |
| 70 | 57528407 | leucine-rich alpha-2-glycoprotein 1 |
| 71 | 149061634 | leucine-rich and death domain containing (predicted) |
| 72 | 153945844 | leukocyte immunoglobulin-like receptor |
| 73 | 13162312 | lipocalin 5 |
| 74 | 37361884 | LRRGT00099 |
| 75 | 157823059 | magnesium-dependent phosphatase 1 |
| 76 | 157820143 | mediator complex subunit 15 |
| 77 | 6981210 | membrane metallo endopeptidase |
| 78 | 22086287 | MHC class I RT1-Au heavy chain precursor |
| 79 | 3006076 | MHC class Ib alpha chain |
| 80 | 2780408 | MIPP65 |
| 81 | 13540689 | moesin |
| 82 | 149038249 | MON1 homolog b (yeast) (predicted), isoform CRA_a |
| 83 | 157822177 | multimerin 2 |
| 84 | 157817871 | multiple EGF-like-domains 9 |
| 85 | 50657404 | murinoglobulin 2 |
| 86 | 3978264 | myosin heavy chain |
| 87 | 149019167 | myosin VC (predicted), isoform CRA_b |
| 88 | 58865810 | N-acetyl galactosaminidase, alpha |
| 89 | 6981260 | NADH dehydrogenase (ubiquinone) 1 alpha subcomplex |
| 90 | 56606108 | NADH dehydrogenase (ubiquinone) Fe-S protein 7 |
| 91 | 77736539 | neuronal pentraxin II |
| 92 | 33516919 | NOVA1_RAT RNA-binding protein Nova-1 (Neuro-oncological ve |
| 93 | 25742576 | nuclear RNA export factor 1 |
| 94 | 47577955 | olfactory receptor Olr1118 |
| 95 | 47577737 | olfactory receptor Olr1261 |
| 96 | 47576017 | olfactory receptor Olr1443 |
| 97 | 47576013 | olfactory receptor Olr1448 |
| 98 | 47576501 | olfactory receptor Olr880 |
| 99 | 6981438 | parathyroid hormone-like peptide |
| 100 | 12018314 | phosphorylase kinase alpha 1 |
| 101 | 13928880 | plasma glutamate carboxypeptidase |
| 102 | 58865988 | pleckstrin homology-like domain, family A, membe |
| 103 | 157817927 | polyhomeotic like 3 |
| 104 | 157787060 | postmeiotic segregation increased 2 |
| 105 | 149058151 | POU domain, class 2, transcription factor 1, isoform |
| 106 | 109464581 | PREDICTED: hypothetical protein |
| 107 | 109472119 | PREDICTED: hypothetical protein |
| 108 | 109472824 | PREDICTED: hypothetical protein |
| 109 | 109475913 | PREDICTED: hypothetical protein |
| 110 | 109496768 | PREDICTED: hypothetical protein |
| 111 | 109507131 | PREDICTED: hypothetical protein |
| 112 | 109510710 | PREDICTED: hypothetical protein |
| 113 | 109465806 | PREDICTED: similar to [Segment 1 of 2] Versican |
| 114 | 109475638 | PREDICTED: similar to absent in melanoma 1 |
| 115 | 109509630 | PREDICTED: similar to armadillo repeat containing |
| 116 | 109459333 | PREDICTED: similar to ATP-binding cassette transpo |
| 117 | 109459785 | PREDICTED: similar to basic leucine zipper transcr |
| 118 | 62655974 | PREDICTED: similar to C1q and tumor necrosis factor |
| 119 | 34851208 | PREDICTED: similar to Cadherin-11 precursor (Osteob |
| 120 | 109475792 | PREDICTED: similar to Carbonic anhydrase 6 precurs |
| 121 | 62641419 | PREDICTED: similar to Catechol O-methyltransferase |
| 122 | 109472681 | PREDICTED: similar to cDNA sequence BC048546 |
| 123 | 109464782 | PREDICTED: similar to CG10011-PA |
| 124 | 109464199 | PREDICTED: similar to CG14085-PB, isoform B |
| 125 | 62648739 | PREDICTED: similar to CG14803-PA |
| 126 | 109464737 | PREDICTED: similar to CG15105-PA, isoform A |
| 127 | 109492014 | PREDICTED: similar to CG7896-PA isoform 2 |
| 128 | 109509326 | PREDICTED: similar to Collagen alpha-1(VI) chain p |
| 129 | 109468076 | PREDICTED: similar to Complement C5 precursor (Hem |
| 130 | 109475425 | PREDICTED: similar to CTP synthase (UTP--ammonia l |
| 131 | 109485723 | PREDICTED: similar to cullin 7 isoform 2 |
| 132 | 109468692 | PREDICTED: similar to cytosolic phospholipase A |
| 133 | 109487395 | PREDICTED: similar to Dedicator of cytokinesis |
| 134 | 34854149 | PREDICTED: similar to ELOVL family member 7, elonga |
| 135 | 109501906 | PREDICTED: similar to F11C1.5a |
| 136 | 109512150 | PREDICTED: similar to Fibronectin type-III doma |
| 137 | 109468368 | PREDICTED: similar to fibrous sheath interactin |
| 138 | 109468797 | PREDICTED: similar to Fibulin-5 precursor (FIBL-5) |
| 139 | 109505720 | PREDICTED: similar to forkhead box F2 |
| 140 | 62641537 | PREDICTED: similar to forkhead box I2 |
| 141 | 109481101 | PREDICTED: similar to G2 and S phase expressed pro |
| 142 | 109507264 | PREDICTED: similar to High mobility group prote |
| 143 | 109494021 | PREDICTED: similar to Interferon-alpha/beta rec |
| 144 | 109480941 | PREDICTED: similar to KIAA1875 protein |
| 145 | 109472587 | PREDICTED: similar to killer cell lectin-like rece |
| 146 | 109458849 | PREDICTED: similar to makorin, ring finger protein |
| 147 | 109489571 | PREDICTED: similar to MKL/myocardin-like 2 |
| 148 | 109460049 | PREDICTED: similar to myoferlin isoform b |
| 149 | 109488330 | PREDICTED: similar to myosin, heavy polypeptide |
| 150 | 109505096 | PREDICTED: similar to Nidogen-1 precursor (Entacti |
| 151 | 109480849 | PREDICTED: similar to NIK and IKK(beta) binding pr |
| 152 | 62639275 | PREDICTED: similar to nonspecific cytotoxic cell re |
| 153 | 109468017 | PREDICTED: similar to nucleoporin 214kDa |
| 154 | 62654060 | PREDICTED: similar to pad-1-like isoform 1 |
| 155 | 34851433 | PREDICTED: similar to papillomavirus L2 interacting |
| 156 | 109483192 | PREDICTED: similar to parathyroid hormone-responsi |
| 157 | 109502759 | PREDICTED: similar to Peptide chain release fac |
| 158 | 109474895 | PREDICTED: similar to procollagen, type XV |
| 159 | 109489144 | PREDICTED: similar to proline-rich protein |
| 160 | 109458623 | PREDICTED: similar to Protein C9orf82 |
| 161 | 109468421 | PREDICTED: similar to Protein KIAA0652 |
| 162 | 109514205 | PREDICTED: similar to putative pheromone recept |
| 163 | 109465346 | PREDICTED: similar to receptor-interacting factor |
| 164 | 109471843 | PREDICTED: similar to sarcolemma associated protei |
| 165 | 62653747 | PREDICTED: similar to Semaphorin-7A precursor (Sema |
| 166 | 109457874 | PREDICTED: similar to serine/threonine kinase |
| 167 | 109468993 | PREDICTED: similar to serine/threonine kinase |
| 168 | 109511167 | PREDICTED: similar to SH3 protein expressed in lym |
| 169 | 109480979 | PREDICTED: similar to slit homolog 1 |
| 170 | 109492830 | PREDICTED: similar to SON protein |
| 171 | 109499378 | PREDICTED: similar to spermatogenesis associated g |
| 172 | 109509473 | PREDICTED: similar to storkhead box 1 |
| 173 | 109460086 | PREDICTED: similar to TATA-binding protein-associa |
| 174 | 109489191 | PREDICTED: similar to testis expressed gene 2 |
| 175 | 109464500 | PREDICTED: similar to tetratricopeptide repeat dom |
| 176 | 109476159 | PREDICTED: similar to transmembrane protein 64 |
| 177 | 109470696 | PREDICTED: similar to ubiquitin protein ligase |
| 178 | 109489413 | PREDICTED: similar to ubiquitin-conjugating enzyme |
| 179 | 109519088 | PREDICTED: similar to Urinary protein 3 precurs |
| 180 | 109474242 | PREDICTED: similar to von Willebrand factor |
| 181 | 109457900 | PREDICTED: similar to WD repeat domain 27 |
| 182 | 109504605 | PREDICTED: similar to Zinc finger CCHC domain-c |
| 183 | 109484074 | PREDICTED: similar to Zinc finger CW-type PWWP |
| 184 | 62653834 | PREDICTED: similar to zinc finger protein 609 |
| 185 | 109464760 | PREDICTED: similar to zinc finger, BED domain c |
| 186 | 9506847 | probasin |
| 187 | 149053219 | profilin 1, isoform CRA_b |
| 188 | 206413 | proline-rich protein |
| 189 | 6981324 | prolyl 4-hydroxylase, beta polypeptide |
| 190 | 56966 | prostatic 22kDa glycoprotein |
| 191 | 29126234 | prostatic steroid binding protein C1 |
| 192 | 160333621 | prostatic steroid-binding protein C2 |
| 193 | 54019434 | protocadherin alpha 2 |
| 194 | 71143096 | protocadherin gamma subfamily C, 3 |
| 195 | 16758172 | quiescin Q6 sulfhydryl oxidase 1 isoform B |
| 196 | 149028467 | rCG22866, isoform CRA_b |
| 197 | 149029308 | rCG23064 |
| 198 | 149018156 | rCG25948 |
| 199 | 149025922 | rCG28698 |
| 200 | 149020590 | rCG31855 |
| 201 | 149054320 | rCG34321, isoform CRA_a |
| 202 | 149050108 | rCG36981 |
| 203 | 149032343 | rCG39091 |
| 204 | 149068411 | rCG39936 |
| 205 | 149027663 | rCG47024 |
| 206 | 149028697 | rCG47051 |
| 207 | 149062421 | rCG47487, isoform CRA_b |
| 208 | 149043237 | rCG50929 |
| 209 | 149045826 | rCG54779 |
| 210 | 149021170 | rCG55947 |
| 211 | 149028518 | rCG55970 |
| 212 | 149040353 | rCG57786 |
| 213 | 149066425 | rCG60008, isoform CRA_a |
| 214 | 149052953 | rCG63502 |
| 215 | 149049666 | rCG64331 |
| 216 | 27465527 | regenerating islet-derived 3 gamma |
| 217 | 149038660 | regulatory factor X domain containing 1 (predicted), |
| 218 | 71681025 | RGD1311906 protein |
| 219 | 13592055 | ribosomal protein L13 |
| 220 | 157819757 | ring finger protein 182 |
| 221 | 6755382 | RuvB-like protein 2 |
| 222 | 157822417 | sal-like 2 |
| 223 | 71043223 | SALM1 |
| 224 | 42627889 | secretoglobin, family 2A, member 2 precursor |
| 225 | 58865630 | serine (or cysteine) peptidase inhibitor, clade |
| 226 | 29293811 | serine (or cysteine) proteinase inhibitor, clade F, |
| 227 | 114326177 | serine hydroxymethyltransferase 1 (soluble) |
| 228 | 157821467 | SH3 domain binding glutamic acid-rich protein-l |
| 229 | 8394373 | signal transducer and activator of transcription 5A |
| 230 | 75516473 | Similar to Cystatin S precursor (LM protein) |
| 231 | 149045435 | similar to mKIAA1429 protein (predicted), isoform CR |
| 232 | 149022854 | similar to RIKEN cDNA 0610027B03, isoform CRA_a |
| 233 | 149022732 | similar to RIKEN cDNA 2600010E01 |
| 234 | 157821187 | SLIT and NTRK-like family, member 2 |
| 235 | 6579191 | SLIT-2 |
| 236 | 57257 | SMR1 protein |
| 237 | 8393886 | solute carrier family 22 member 6 |
| 238 | 13928920 | solute carrier family 28, member 2 |
| 239 | 8394200 | solute carrier family 6 (neurotransmitter transporte |
| 240 | 157819145 | SPARC related modular calcium binding 2 |
| 241 | 6981502 | spermine binding protein |
| 242 | 135072 | SVS4_RAT Seminal vesicle secretory protein 4 precursor (Se |
| 243 | 56912221 | thioredoxin interacting protein |
| 244 | 149061031 | thymocyte selection-associated HMG box gene (predict |
| 245 | 25742799 | toll-like receptor 4 |
| 246 | 77695935 | transforming growth factor, beta receptor 1 |
| 247 | 145312243 | transglutaminase 4 (prostate) |
| 248 | 157817741 | transmembrane channel-like 2 |
| 249 | 12711700 | tRNA selenocysteine associated protein 1 |
| 250 | 6981672 | tropomyosin 4 |
| 251 | 82654194 | troponin C2, fast |
| 252 | 4507761 | ubiquitin and ribosomal protein L40 precursor |
| 253 | 13929126 | UDP-N-acetyl-alpha-D-galactosamine:polypeptide N-ac |
| 254 | 11559982 | unc-5 homolog B |
| 255 | 71051741 | Unknown (protein for IMAGE:7377044) |
| 256 | 165971057 | Unknown (protein for MGC:188080) |
| 257 | 187469128 | Unknown (protein for MGC:188922) |
| 258 | 56854 | unnamed protein product |
| 259 | 57406 | unnamed protein product |
| 260 | 6226288 | UP1_RAT Urinary protein 1 precursor (RUP-1) (Liver regenera |
| 261 | 71043730 | vanin 1 |
| 262 | 157820363 | vanin 3 |
| 263 | 46397078 | VIGLN_RAT Vigilin (High density lipoprotein-binding protei |
| 264 | 52138521 | villin 2 |
| 265 | 57114230 | vomeronasal 1 receptor, g8 |
| 266 | 153792782 | vomeronasal 2 receptor 50 |
| 267 | 157819553 | WW, C2 and coiled-coil domain containing 2 |
| 268 | 149025116 | zinc binding alcohol dehydrogenase, domain containin |
| 269 | 66730327 | zinc finger protein 458 |
| 270 | 58865790 | zinc finger protein 513 |
| 271 | 157823013 | zinc finger protein 775 |
| 272 | 58865922 | zinc finger, AN1-type domain 3 |

| **Table S1-d: 180 Proteins unique to Infected female rat urine** | | |
| --- | --- | --- |
| **Sl #** | **Gene ID** | **Name of the Protein** |
| 1 | 187469191 | Paip1_predicted protein |
| 2 | 110287990 | A Chain A, Crystal Structure Of An Aspartoacylase From Rat |
| 3 | 13399336 | AAK31339 KIAA1454-like protein |
| 4 | 50233797 | Abelson helper integration site 1 |
| 5 | 39930499 | acid alpha-glucosidase |
| 6 | 6900796 | adducin 2 (beta) isoform b |
| 7 | 77539436 | AHNAK 1 |
| 8 | 115720 | alpha-1-inhibitor III precursor |
| 9 | 157821019 | ash2 (absent, small, or homeotic)-like |
| 10 | 68341963 | asparagine-linked glycosylation 5 homolog (yeast |
| 11 | 157820121 | AT rich interactive domain 4A (Rbp1 like) |
| 12 | 27465565 | ATP-binding cassette, sub-family A (ABC1), member 5 |
| 13 | 157817498 | basonuclin 2 |
| 14 | 20806135 | beta-defensin 22 |
| 15 | 157786982 | carbonic anhydrase 15 |
| 16 | 114145712 | CARM1_RAT Histone-arginine methyltransferase CARM1 (Prote |
| 17 | 6433984 | Cas-associated zinc finger protein |
| 18 | 157786930 | complement component 1, q subcomponent-like 2 |
| 19 | 40018606 | cyclic nucleotide-gated cation channel beta 3 |
| 20 | 157818855 | cyclin M3 |
| 21 | 157821569 | DEP domain containing 2 |
| 22 | 50511338 | diacylglycerol lipase, alpha |
| 23 | 71051338 | DLGP3_RAT Disks large-associated protein 3 (DAP-3) (SAP90/ |
| 24 | 61557109 | DNA polymerase lambda |
| 25 | 6978449 | elastase 2A |
| 26 | 157818293 | envoplakin |
| 27 | 27465533 | epididymal secretory protein E1 |
| 28 | 149058512 | excision repair cross-complementing rodent repair de |
| 29 | 157820197 | fer-1-like 4 |
| 30 | 50511316 | fucosyltransferase 8 |
| 31 | 16758244 | gamma-aminobutyric acid (GABA) A receptor, gamma 1 |
| 32 | 157817127 | glycine decarboxylase |
| 33 | 34854062 | GRID2_RAT Glutamate receptor delta-2 subunit precursor (Gl |
| 34 | 58866004 | guanine nucleotide binding protein, alpha 14 |
| 35 | 11177910 | guanylate cyclase 2e |
| 36 | 9507119 | heat shock protein 2 |
| 37 | 6978803 | hemoglobin alpha 1 chain |
| 38 | 77628163 | hydroxysteroid 11-beta dehydrogenase 1 |
| 39 | 57526868 | hypothetical protein LOC291784 |
| 40 | 157818227 | hypothetical protein LOC291853 |
| 41 | 66730425 | hypothetical protein LOC294883 |
| 42 | 114145602 | hypothetical protein LOC296384 |
| 43 | 110590413 | hypothetical protein LOC297832 |
| 44 | 68163387 | hypothetical protein LOC497888 |
| 45 | 114145407 | hypothetical protein LOC498354 |
| 46 | 65301416 | hypothetical protein LOC498873 |
| 47 | 157822415 | hypothetical protein LOC500233 |
| 48 | 6729087 | immunoglobulin heavy chain variable region |
| 49 | 6900770 | immunoglobulin heavy chain variable region |
| 50 | 149068632 | immunoglobulin heavy chain variable region |
| 51 | 202577 | immunoglobulin light chain |
| 52 | 4506635 | immunoglobulin light chain variable region |
| 53 | 47577889 | keratin 5 |
| 54 | 13638436 | keratin 7 |
| 55 | 14861854 | kinesin family member 3C |
| 56 | 78214365 | kininogen 1 |
| 57 | 157817688 | laminin, beta 1 |
| 58 | 38372261 | LanC-like 1 |
| 59 | 20301982 | lectin, galactoside-binding, soluble, 3 binding pro |
| 60 | 149041963 | leucine rich repeat containing 23, isoform CRA_c |
| 61 | 157823978 | liver-expressed antimicrobial peptide 2 |
| 62 | 70794776 | LOC362795 protein |
| 63 | 157823569 | mediator complex subunit 16 |
| 64 | 157818245 | methylmalonic aciduria (cobalamin deficiency) c |
| 65 | 18034793 | Munc13-4 protein |
| 66 | 27151789 | myosin IE |
| 67 | 21902531 | Na/Pi cotransporter 4 |
| 68 | 204706 | neu-related lipocalin, NRL=SV40-induced 24p3 gene prod |
| 69 | 157819315 | NIMA (never in mitosis gene a)-related expresse |
| 70 | 47577559 | olfactory receptor Olr1162 |
| 71 | 47155567 | olfactory receptor Olr1490 |
| 72 | 47576613 | olfactory receptor Olr1767 |
| 73 | 47576743 | olfactory receptor Olr190 |
| 74 | 47576795 | olfactory receptor Olr379 |
| 75 | 47577235 | olfactory receptor Olr446 |
| 76 | 47577395 | olfactory receptor Olr514 |
| 77 | 47575943 | olfactory receptor Olr85 |
| 78 | 6981010 | opioid receptor, delta 1 |
| 79 | 157819155 | oxysterol binding protein-like 11 |
| 80 | 187281866 | Paip1_predicted protein |
| 81 | 2293468 | PAM COOH-terminal interactor protein 10b |
| 82 | 55778372 | PAS domain containing serine/threonine kinase |
| 83 | 149059302 | phosphatidylethanolamine binding protein 1, isoform |
| 84 | 62079061 | pre-mRNA cleavage factor I, 59 kDa subunit |
| 85 | 109461450 | PREDICTED: hypothetical protein |
| 86 | 109469097 | PREDICTED: hypothetical protein |
| 87 | 109483130 | PREDICTED: hypothetical protein |
| 88 | 109472532 | PREDICTED: similar to AT rich interactive domain 3 |
| 89 | 109458947 | PREDICTED: similar to ATP-binding cassette transpo |
| 90 | 109498339 | PREDICTED: similar to ATPase, aminophospholipid tr |
| 91 | 109484648 | PREDICTED: similar to axonemal dynein heavy cha |
| 92 | 109468939 | PREDICTED: similar to centrosomal protein 2 isofor |
| 93 | 109468089 | PREDICTED: similar to CG13531-PB |
| 94 | 109459335 | PREDICTED: similar to Cytochrome P450 2B1 (CYPI |
| 95 | 109465790 | PREDICTED: similar to DENN/MADD domain containi |
| 96 | 109508238 | PREDICTED: similar to double homeobox 4c |
| 97 | 109462744 | PREDICTED: similar to FAT tumor suppressor homolog |
| 98 | 109510643 | PREDICTED: similar to GTPase activating protein te |
| 99 | 109468725 | PREDICTED: similar to insulinoma-associated 1 |
| 100 | 109497830 | PREDICTED: similar to isoleucine-tRNA synthetase 2 |
| 101 | 109481069 | PREDICTED: similar to keratin Kb40 |
| 102 | 109483482 | PREDICTED: similar to KIAA0999 protein |
| 103 | 109458640 | PREDICTED: similar to kinesin family member 7 |
| 104 | 109495868 | PREDICTED: similar to Lactase-phlorizin hydrolase |
| 105 | 62650542 | PREDICTED: similar to low density lipoprotein-relat |
| 106 | 109495000 | PREDICTED: similar to M-phase phosphoprotein 9 |
| 107 | 62638565 | PREDICTED: similar to missing oocyte CG7074-PA |
| 108 | 109469717 | PREDICTED: similar to Murinoglobulin 1 homolog |
| 109 | 109464786 | PREDICTED: similar to Myosin-4 (Myosin heavy ch |
| 110 | 109487139 | PREDICTED: similar to N-sulfoglucosamine sulfoh |
| 111 | 109481333 | PREDICTED: similar to olfactory receptor Olr116 |
| 112 | 109461750 | PREDICTED: similar to PI-3-kinase-related kinas |
| 113 | 109492339 | PREDICTED: similar to Protein KIAA1543 |
| 114 | 62079221 | PREDICTED: similar to serine/threonine kinase |
| 115 | 109474829 | PREDICTED: similar to tetratricopeptide repeat dom |
| 116 | 62646850 | PREDICTED: similar to tetratricopeptide repeat doma |
| 117 | 27545388 | PREDICTED: similar to Thrombospondin-2 precursor |
| 118 | 109467336 | PREDICTED: similar to Tubulin--tyrosine ligase- |
| 119 | 109499663 | PREDICTED: similar to vitamin A-deficient testi |
| 120 | 109477909 | PREDICTED: similar to WW domain binding protein |
| 121 | 109509542 | PREDICTED: similar to WW, C2 and coiled-coil domai |
| 122 | 109504721 | PREDICTED: similar to Zinc finger protein 469 |
| 123 | 84794625 | PREDICTED: similar to zinc finger protein 91 |
| 124 | 71153510 | progestin and adipoQ receptor family member VII |
| 125 | 80861401 | protein phosphatase 2C, magnesium dependent, cat |
| 126 | 58476521 | protocadherin gamma subfamily B, 7 |
| 127 | 3135078 | PTPRN_RAT Receptor-type tyrosine-protein phosphatase-like N |
| 128 | 157817652 | PWWP domain containing 2 |
| 129 | 13242283 | rabaptin, RAB GTPase binding effector protein 2 |
| 130 | 2317896 | RAG1 |
| 131 | 4099051 | RAP1A, member of RAS oncogene family |
| 132 | 149034095 | rCG21055 |
| 133 | 149063978 | rCG28025, isoform CRA_a |
| 134 | 149065309 | rCG28346 |
| 135 | 149016558 | rCG30666, isoform CRA_a |
| 136 | 149049478 | rCG34176, isoform CRA_d |
| 137 | 149065352 | rCG40022 |
| 138 | 149027661 | rCG40905 |
| 139 | 149032734 | rCG40944 |
| 140 | 149029984 | rCG42124 |
| 141 | 149033044 | rCG42268 |
| 142 | 149056175 | rCG46528 |
| 143 | 149034301 | rCG51065, isoform CRA_a |
| 144 | 149038370 | rCG58330 |
| 145 | 149025882 | rCG63063 |
| 146 | 149053781 | rCG63520 |
| 147 | 149034286 | rCG64219 |
| 148 | 46310098 | Rho GTPase activating protein 20 |
| 149 | 4506413 | ribosomal protein L32 |
| 150 | 149063507 | ring finger protein 31 (predicted), isoform CRA_a |
| 151 | 13591758 | S10A9_RAT Protein S100-A9 (S100 calcium-binding protein A9 |
| 152 | 24025621 | S12A3_RAT Solute carrier family 12 member 3 (Thiazide-sens |
| 153 | 62652351 | SA hypertension-associated homolog |
| 154 | 6981558 | Sec61 alpha 1 subunit |
| 155 | 58865998 | serine (or cysteine) peptidase inhibitor, clade |
| 156 | 1326433 | SH3p4 |
| 157 | 68163501 | similar to constitutive photomorphogenic protein |
| 158 | 149024343 | similar to KIAA1627 protein (predicted), isoform CRA |
| 159 | 57526933 | Similar to pancreatitis-induced protein 49 |
| 160 | 7019415 | sodium channel, voltage-gated, type X, alpha |
| 161 | 157820109 | solute carrier family 12, member 6 |
| 162 | 8394242 | solute carrier family 22, member 5 |
| 163 | 157821955 | solute carrier family 25, member 41 |
| 164 | 157820817 | solute carrier family 26, member 9 |
| 165 | 157819907 | solute carrier family 43, member 1 |
| 166 | 157823837 | solute carrier family 5, member 4a |
| 167 | 6981522 | solute carrier family 9 (sodium/hydrogen exchanger), |
| 168 | 157787056 | suppressor of cytokine signaling 5 |
| 169 | 6981308 | syndecan 4 |
| 170 | 21717797 | syntaxin 17 |
| 171 | 57164121 | T-kininogen II precursor |
| 172 | 186910243 | tigger transposable element derived 5 |
| 173 | 3183542 | titin |
| 174 | 109513719 | TMM27_RAT Collectrin precursor (Transmembrane protein 27) |
| 175 | 54312094 | Tripartite motif protein 35 |
| 176 | 55715693 | Tspan6 protein |
| 177 | 114149244 | ubiquitin-conjugating enzyme E2M (UBC12 homolog, yea |
| 178 | 150187543 | unc-119 homolog B |
| 179 | 149055540 | zinc finger protein 507 (predicted), isoform CRA_a |
| 180 | 157820907 | zonadhesin |

| **Table S1-e: 39 Proteins common to control and female lepto rat urine: absent in infected males** | | |
| --- | --- | --- |
| **Sl #** | **Gene ID** | **Name of the Protein** |
| 1 | 609246 | alpha-1 proteinase inhibitor 3 |
| 2 | 22219448 | alpha-2u globulin PGCL3 |
| 3 | 57527131 | ARP10 actin-related protein 10 homolog ARP10 act |
| 4 | 758260 | beta-glucuronidase precursor |
| 5 | 29789038 | bone morphogenetic protein 6 |
| 6 | 13431334 | CADH2_RAT Cadherin-2 precursor (Neural cadherin) (N-cadh |
| 7 | 157817294 | caspase 8 associated protein 2 |
| 8 | 8393218 | cathepsin C |
| 9 | 62079239 | CDC-like kinase 2 |
| 10 | 3219176 | CDP-diacylglycerol synthase |
| 11 | 51948462 | endothelial cell adhesion molecule |
| 12 | 25742617 | epidermal growth factor receptor |
| 13 | 157818407 | exocyst complex component 3-like |
| 14 | 149015981 | fibronectin 1, isoform CRA_d |
| 15 | 62945264 | hypothetical protein LOC307833 |
| 16 | 53850644 | hypothetical protein LOC313430 |
| 17 | 157820367 | hypothetical protein LOC691352 |
| 18 | 7650268 | immunoglobulin kappa light chain variable region |
| 19 | 7650288 | immunoglobulin kappa light chain variable region |
| 20 | 2292988 | Inter-alpha-inhibitor H4 heavy chain |
| 21 | 52851385 | kallikrein 1 |
| 22 | 42627893 | major urinary protein 5 |
| 23 | 149067059 | mannosyl (alpha-1,3-)-glycoprotein beta-1,4-N-acetyl |
| 24 | 47575951 | olfactory receptor Olr1498 |
| 25 | 19705509 | polyamine modulated factor 1 binding protein 1 |
| 26 | 109480255 | PREDICTED: similar to Calponin-2 (Calponin H2, |
| 27 | 109489481 | PREDICTED: similar to chromosome 17 open reading f |
| 28 | 109485899 | PREDICTED: similar to chromosome 9 open reading |
| 29 | 109483719 | PREDICTED: similar to Collagen alpha-1(XII) chain |
| 30 | 109478606 | PREDICTED: similar to Endoribonuclease Dicer (D |
| 31 | 109491389 | PREDICTED: similar to myosin XVIIIa |
| 32 | 149022078 | rCG27110 |
| 33 | 149066845 | rCG49069 |
| 34 | 40807349 | receptor interacting protein kinase 5 |
| 35 | 149041027 | similar to RIKEN cDNA B130052G07 (predicted), isofor |
| 36 | 6981568 | superoxide dismutase 3, extracellular |
| 37 | 149048680 | tetratricopeptide repeat domain 14 (predicted), isof |
| 38 | 171847082 | Unknown (protein for MGC:187760) |
| 39 | 165970905 | Unknown (protein for MGC:188615) |

| **Table S1-f: 18 Proteins common to control animals & male lepto rat urine: absent in infected females** | | |
| --- | --- | --- |
| **Sl #** | **Gene ID** | **Name of the protein** |
| 1 | 149052278 | arginyl-tRNA synthetase (predicted), isoform CRA_a |
| 2 | 47059181 | complement factor B |
| 3 | 157819707 | cytochrome P450, family 2, subfamily s, polypep |
| 4 | 149039175 | DEAD/H (Asp-Glu-Ala-Asp/His) box polypeptide 31 (pre |
| 5 | 157821779 | dynactin 3 |
| 6 | 55926190 | eosinophil-associated, ribonuclease A family, me |
| 7 | 33303718 | erythroid spectrin alpha |
| 8 | 231468 | FETUA_RAT Alpha-2-HS-glycoprotein precursor (Fetuin-A) (Glyc |
| 9 | 157822545 | HEAT repeat containing 1 |
| 10 | 604902 | Kan-1 |
| 11 | 27465585 | keratin 20 |
| 12 | 157818899 | NFKB inhibitor interacting Ras-like protein 1 |
| 13 | 109472233 | PREDICTED: similar to CG1965-PA |
| 14 | 75517000 | Prp-5 protein |
| 15 | 149027664 | rCG64161 |
| 16 | 58866012 | tripartite motif-containing 55 |
| 17 | 6756041 | tyrosine 3-monooxygenase/tryptophan 5-monooxygenase |
| 18 | 162287178 | vitronectin |

| **Table S1-g: 51 Proteins common to male and female lepto rat urine: absent in control animals.** | | |
| --- | --- | --- |
| **Sl #** | **Gene ID** | **Name of the protein** |
| 1 | 13540691 | 5-methyltetrahydrofolate-homocysteine methyltransfe |
| 2 | 4501881 | alpha 1 actin precursor |
| 3 | 8347066 | aminopeptidase |
| 4 | 54124354 | anthrax toxin receptor; tumor endothelial marker 8 |
| 5 | 50812707 | anti-mouse IL-4 specific monoclonal antibody 11B11 li |
| 6 | 58865670 | biotinidase |
| 7 | 468766 | carboxylesterase; serum carboxylesterase |
| 8 | 149020682 | centrosomal protein 57, isoform CRA_a |
| 9 | 6978695 | ceruloplasmin |
| 10 | 13591934 | chitobiase, di-N-acetyl- |
| 11 | 157818907 | chromobox homolog 2 |
| 12 | 157821559 | elastase 3B, pancreatic |
| 13 | 58865654 | epidermal growth factor-containing fibulin-like |
| 14 | 59676599 | family with sequence similarity 20, member C |
| 15 | 157823535 | fucokinase |
| 16 | 157824103 | galactosidase, beta 1 |
| 17 | 6978884 | glucokinase regulatory protein |
| 18 | 62078949 | glypican 4 |
| 19 | 60097941 | haptoglobin |
| 20 | 68226711 | hypothetical protein LOC310926 |
| 21 | 6900535 | immunoglobulin heavy chain variable region |
| 22 | 7414591 | immunoglobulin heavy chain variable region |
| 23 | 7650282 | immunoglobulin kappa light chain variable region |
| 24 | 81295359 | jumonji domain containing 5 |
| 25 | 38304001 | Karyopherin (importin) alpha 2 |
| 26 | 112983842 | La ribonucleoprotein domain family, member 7 |
| 27 | 56972100 | LOC299567 protein |
| 28 | 56788960 | LOC367586 protein |
| 29 | 16758210 | nucleobindin 1 |
| 30 | 16758216 | plasminogen |
| 31 | 109475195 | PREDICTED: hypothetical protein |
| 32 | 109480474 | PREDICTED: hypothetical protein |
| 33 | 109498116 | PREDICTED: similar to ezrin-binding partner PACE-1 |
| 34 | 109460053 | PREDICTED: similar to Gastric triacylglycerol lipa |
| 35 | 109501207 | PREDICTED: similar to nidogen 2 |
| 36 | 109480300 | PREDICTED: similar to peptidoglycan recognition |
| 37 | 109478477 | PREDICTED: similar to regulator of G-protein si |
| 38 | 109501286 | PREDICTED: similar to Tu translation elongation |
| 39 | 109483261 | PREDICTED: similar to Urinary protein 3 precurs |
| 40 | 149027666 | rCG47026 |
| 41 | 149037311 | rCG53368 |
| 42 | 149059573 | rCG55184, isoform CRA_a |
| 43 | 149037293 | rCG64259 |
| 44 | 149037296 | rCG64262 |
| 45 | 111977 | S25644 Ig mu chain C region - rat (fragment) |
| 46 | 19173736 | serine carboxypeptidase 1 |
| 47 | 68052911 | SPA3M_RAT Serine protease inhibitor A3M precursor (Serpin |
| 48 | 157817049 | tetratricopeptide repeat domain 25 |
| 49 | 61556986 | transferrin |
| 50 | 438880 | tropomyosin |
| 51 | 267251 | UROK_RAT Urokinase-type plasminogen activator precursor (uPA |
