# Supplementary material for: Proteomic Analysis of Urine Exosomes Reveals Renal Tubule Response to Leptospiral Colonization in Experimentally Infected Rats
Source: PLoS Negl Trop Dis. 2015 Mar 20;9(3):e0003640. doi: 10.1371/journal.pntd.0003640 (PMC4368819; doi:10.1371/journal.pntd.0003640)
Supplement: S2 Table — b: Proteins unique to control rat urine exosomes, but absent in infected rat urine exosomes. C: Proteins unique to Infected male rat urine exosomes, but absent in control animals and infected female rat samples. (DOCX) [file pntd.0003640.s007.docx]

| **Table S2-a. Proteins commonly present in urinary exosomes of all groups:**  **Uninfected, infected male and infected female rats** | | |
| --- | --- | --- |
| **Sl #** | **Gene Id** | **Name of the protein** |
| 1 | 13592133 | actin, cytoplasmic 1 |
| 2 | 17902245 | AF450298_1 EZRIN |
| 3 | 158267591 | chloride intracellular channel 1 |
| 4 | 10198602 | collectrin precursor |
| 5 | 14010871 | dipeptidyl peptidase 2 precursor |
| 6 | 158081751 | fructose-bisphosphate aldolase B |
| 7 | 83026049 | glutamate cysteine ligase modifier subunit |
| 8 | 25742748 | glutamate--cysteine ligase catalytic subunit |
| 9 | 140970581 | glutamyl aminopeptidase |
| 10 | 300797670 | G-protein coupled receptor family C group 5 member C precursor |
| 11 | 51980580 | Meprin 1 beta |
| 12 | 11024674 | Na(+)/H(+) exchange regulatory cofactor NHE-RF1 |
| 13 | 13928998 | Na(+)/H(+) exchange regulatory cofactor NHE-RF3 |
| 14 | 6981210 | neprilysin |
| 15 | 149037632 | procollagen, type VI, alpha 3 (predicted), isoform CRA_d |
| 16 | 57231 | unnamed protein product |
| 17 | 157817658 | villin-1 |
| 18 | 203451 | water channel |
| 19 | 149033753 | albumin, isoform CRA_a |
| 20 | 149035585 | aldo-keto reductase family 1, member A1, isoform CRA_b |
| 21 | 6981420 | anionic trypsin-1 precursor |
| 22 | 6978769 | deoxyribonuclease-1 precursor |
| 23 | 24637713 | dermcidin precursor |
| 24 | 149022111 | dipeptidylpeptidase 4, isoform CRA_a |
| 25 | 403949907 | epidermal growth factor |
| 26 | 149043754 | gamma-glutamyltransferase 1, isoform CRA_b |
| 27 | 12083635 | histone H4 |
| 28 | 984680 | kappa immunoglobulin |
| 29 | 38303961 | Keratin 5 |
| 30 | 57012436 | keratin, type I cytoskeletal 10 |
| 31 | 56912233 | keratin, type I cytoskeletal 14 |
| 32 | 57012446 | keratin, type I cytoskeletal 42 |
| 33 | 120474989 | keratin, type II cytoskeletal 1 |
| 34 | 57114290 | keratin, type II cytoskeletal 2 epidermal |
| 35 | 57012352 | keratin, type II cytoskeletal 75 |
| 36 | 114145409 | keratin, type II cytoskeletal cochleal |
| 37 | 51980502 | Meprin 1 alpha |
| 38 | 8394307 | neutral and basic amino acid transport protein rBAT |
| 39 | 392354923 | PREDICTED: glyceraldehyde-3-phosphate dehydrogenase-like |
| 40 | 293348969 | PREDICTED: keratin, type II cytoskeletal 6A-like |
| 41 | 392355802 | PREDICTED: transcriptional regulator ATRX, partial |
| 42 | 392333871 | PREDICTED: ubiquitin-40S ribosomal protein S27a-like |
| 43 | 149022164 | rCG26871, isoform CRA_c |
| 44 | 149015740 | rCG39189, isoform CRA_b |
| 45 | 149031968 | rCG50682, isoform CRA_b |
| 46 | 149041392 | rCG57965, isoform CRA_b |
| 47 | 133777069 | Serine (or cysteine) peptidase inhibitor, clade A, member 3K |
| 48 | 58865770 | solute carrier family 7 member 13 |
| 49 | 16924020 | xaa-Pro aminopeptidase 2 precursor |

| **Table S2-b: Proteins Unique to Control rat urine exosomes: Absent in infected rat urine exosomes** | | |
| --- | --- | --- |
| **Sl #** | **Gene Id** | **Name of the protein** |
| 1 | 11127974 | AF314657_1 clusterin, partial |
| 2 | 149020967 | aldo-keto reductase family 1, member C18, isoform CRA_b |
| 3 | 8307696 | alpha-2u globulin |
| 4 | 6978501 | annexin A1 |
| 5 | 119959830 | Beta-actin |
| 6 | 14010887 | calbindin |
| 7 | 158138561 | complement C3 precursor |
| 8 | 38303863 | Egf protein |
| 9 | 20806135 | galectin-3-binding protein precursor |
| 10 | 204501 | glutathione S-transferase (EC 2.5.1.18) |
| 11 | 13928688 | glutathione S-transferase alpha-3 |
| 12 | 2443314 | keratin 14 |
| 13 | 51889726 | keratin, type I cytoskeletal 24 |
| 14 | 205830436 | myosin heavy chain IIa |
| 15 | 106879208 | myosin-4 |
| 16 | 13928928 | napsin A aspartic peptidase precursor |
| 17 | 1345430 | perchrolic acid soluble protein |
| 18 | 206113 | phosphoglycerate kinase |
| 19 | 197313681 | protein FAM54A |
| 20 | 930263 | proteinase inhibitor-like protein (202 AA) |
| 21 | 149020460 | RAB3D, member RAS oncogene family, isoform CRA_b |
| 22 | 149018134 | rCG25711, isoform CRA_d |
| 23 | 149041136 | rCG27526 |
| 24 | 149065349 | rCG28294 |
| 25 | 149052643 | rCG33456, isoform CRA_d |
| 26 | 149042305 | rCG36303 |
| 27 | 149015742 | rCG39189, isoform CRA_c |
| 28 | 149049318 | similar to ovostatin-2 (predicted), isoform CRA_d |
| 29 | 149032801 | solute carrier family 6 (neurotransmitter transporter), member 18, isoform CRA_a |
| 30 | 149068736 | START domain containing 10, isoform CRA_a |
| 31 | 149068448 | tripeptidyl peptidase I, isoform CRA_e |
| 32 | 149031965 | type II keratin Kb1 |

| **Table S2-c: Proteins unique to Infected male rat urine exosomes:**  **absent in control animals and infected female rat samples** | | |
| --- | --- | --- |
| **Sl #** | **Gene Id** | **Name of the protein** |
| 1 | 19071457 | AF446004_1 organic anion transporter K12 |
| 2 | 205384 | alpha-1-macroglobulin |
| 3 | 149028871 | annexin A2, isoform CRA_b |
| 4 | 30023556 | ATP-binding cassette transporter ABCG2 |
| 5 | 84781664 | bifunctional ATP-dependent dihydroxyacetone kinase/FAD-AMP lyase (cyclizing) |
| 6 | 149026325 | cystathionase (cystathionine gamma-lyase) |
| 7 | 203734 | cytokeratin 8 polypeptide |
| 8 | 149045175 | desmoplakin, isoform CRA_b |
| 9 | 257467627 | Fc fragment of IgG binding protein-like precursor |
| 10 | 25742757 | glutathione synthetase |
| 11 | 59803289 | GTP-binding protein G-alpha-i2 splice variant a |
| 12 | 71089915 | guanine nucleotide binding protein alpha inhibiting 3 |
| 13 | 111153966 | H+/organic cation antiporter variant 2 |
| 14 | 89573967 | isocitrate dehydrogenase 1 |
| 15 | 55741540 | katanin p60 ATPase-containing subunit A-like 1 |
| 16 | 157817668 | L-amino acid oxidase 1 precursor |
| 17 | 15100179 | malate dehydrogenase, cytoplasmic |
| 18 | 125664309 | mitochondrial chloride intracellular channel 4 |
| 19 | 149018869 | plastin 1 (I isoform) (predicted), isoform CRA_a |
| 20 | 293342378 | PREDICTED: anoctamin-8-like |
| 21 | 293344702 | PREDICTED: gastric triacylglycerol lipase-like |
| 22 | 392339909 | PREDICTED: uncharacterized protein LOC679818 |
| 23 | 158635966 | prominin 1 isoform 2 precursor |
| 24 | 145312243 | protein-glutamine gamma-glutamyltransferase 4 |
| 25 | 49522647 | Rab8a protein, partial |
| 26 | 56541165 | Rab8a protein, partial |
| 27 | 40804379 | radixin |
| 28 | 149044747 | rCG23301, isoform CRA_a |
| 29 | 149022163 | rCG26871, isoform CRA_b |
| 30 | 149068560 | rCG39881, isoform CRA_a |
| 31 | 149061924 | rCG48611, isoform CRA_c |
| 32 | 149031970 | rCG50690 |
| 33 | 171846576 | Slc5a10 protein |
| 34 | 38512111 | Tpi1 protein, partial |
| 35 | 55825 | unnamed protein product |
| 36 | 530167 | ventral prostate-specific protein |
| 37 | 149018288 | villin-like (predicted), isoform CRA_b |

| **Table S2-d: Proteins unique to Infected female rat urine exosomes: absent in control animals and infected male rats** | | |
| --- | --- | --- |
| **Sl #** | **Gene Id** | **Name of the protein** |
| 1 | 149034976 | actin, beta, isoform CRA_c |
| 2 | 6978441 | actin, gamma-enteric smooth muscle |
| 3 | 149030485 | ATPase, Na+/K+ transporting, alpha 1 polypeptide, isoform CRA_a |
| 4 | 149025671 | guanine nucleotide binding protein, alpha transducing 2 (predicted), isoform CRA_a |
| 5 | 51591909 | keratin, type I cytoskeletal 13 |
| 6 | 57012362 | keratin, type II cytoskeletal 72 |
| 7 | 57012358 | keratin, type II cytoskeletal 73 |
| 8 | 149049040 | lactate dehydrogenase B, isoform CRA_b |
| 9 | 149051482 | methylenetetrahydrofolate dehydrogenase (NADP+ dependent), methenyltetrahydrofolate cyclohydrolase, |
| 10 | 392354563 | PREDICTED: meprin A subunit beta-like, partial |
| 11 | 149065352 | rCG28346 |
| 12 | 149016816 | similar to hypothetical protein BC008207 |
| 13 | 149019049 | similar to Myosin VI (predicted), isoform CRA_b |
| 14 | 148747335 | solute carrier family 22 member 7 |
| 15 | 149039876 | solute carrier family 34 (sodium phosphate), member 1, isoform CRA_d |
| 16 | 149067631 | solute carrier family 5 (sodium/glucose cotransporter), member 2, isoform CRA_a |
| 17 | 197245994 | Susd2 protein |

| **Table S2-e: Proteins common to control and female lepto rat urine exosomes: absent in infected males** | | |
| --- | --- | --- |
| **Sl #** | **Gene Id** | **Name of the protein** |
| 1 | 89242501 | immunoglobulin kappa constant region |
| 2 | 1698704 | mast cell protease 9 |
| 3 | 149056040 | napsin A aspartic peptidase, isoform CRA_b |
| 4 | 109482941 | PREDICTED: keratin, type II cytoskeletal 6A-like isoform 1 |
| 5 | 392347244 | PREDICTED: maltase-glucoamylase, intestinal-like, partial |
| 6 | 149024688 | rCG31027, isoform CRA_b |
| 7 | 149054207 | rCG33578 |
| 8 | 149031961 | rCG50520 |
| 9 | 149031969 | rCG50775 |
| 10 | 601865 | aminopeptidase M |
| 11 | 149056721 | apolipoprotein E, isoform CRA_c |

| **Table S2-f: Proteins common to control animals and male lepto rat urine exosomes: absent in infected female rats** | | |
| --- | --- | --- |
| **Sl #** | **Gene Id** | **Name of the protein** |
| 1 | 6981712 | 14-3-3 protein theta |
| 2 | 1051270 | 14-3-3 zeta isoform |
| 3 | 157823471 | 6-phosphogluconolactonase |
| 4 | 7673035 | AF146518_1 aminopeptidase A short variant |
| 5 | 433611 | aflatoxin B1 aldehyde reductase |
| 6 | 149020189 | aldolase B, isoform CRA_b |
| 7 | 204264 | alpha-2u globulin, partial |
| 8 | 158186649 | alpha-enolase |
| 9 | 13591914 | aminopeptidase N precursor |
| 10 | 56971297 | Amy1a protein |
| 11 | 149066347 | annexin A13 (predicted) |
| 12 | 149026460 | brain abundant, membrane attached signal protein 1, isoform CRA_b |
| 13 | 11560135 | brain acid soluble protein 1 |
| 14 | 149028043 | chloride intracellular channel 1, isoform CRA_b |
| 15 | 149024252 | chloride intracellular channel 4, isoform CRA_a |
| 16 | 392937 | common salivary protein 1 |
| 17 | 56013 | CRP2 |
| 18 | 56030 | cystathionine gamma-lyase |
| 19 | 58219062 | cytosolic non-specific dipeptidase |
| 20 | 818029 | dismutase |
| 21 | 52138521 | ezrin |
| 22 | 8393446 | glutamate--cysteine ligase regulatory subunit |
| 23 | 149030882 | glutathione synthetase, isoform CRA_b |
| 24 | 149066547 | heat-responsive protein 12, isoform CRA_b |
| 25 | 47087085 | keratin, type I cytoskeletal 17 |
| 26 | 155369696 | keratin, type II cytoskeletal 6A |
| 27 | 8393641 | kynurenine/alpha-aminoadipate aminotransferase, mitochondrial |
| 28 | 13540689 | moesin |
| 29 | 149042266 | moesin, isoform CRA_a |
| 30 | 50925471 | PDZ domain containing 1 |
| 31 | 8393910 | phosphatidylethanolamine-binding protein 1 |
| 32 | 20302085 | polyubiquitin-B precursor |
| 33 | 392351673 | PREDICTED: LOW QUALITY PROTEIN: uncharacterized protein LOC287750 |
| 34 | 109466036 | PREDICTED: phosphoglycerate kinase 1-like |
| 35 | 9506847 | probasin precursor |
| 36 | 210032180 | programmed cell death 6-interacting protein |
| 37 | 114055048 | prominin 1 |
| 38 | 40254781 | rab GDP dissociation inhibitor beta |
| 39 | 149019084 | rCG25445, isoform CRA_f |
| 40 | 149018133 | rCG25711, isoform CRA_c |
| 41 | 149018671 | rCG25777, isoform CRA_a |
| 42 | 149041139 | rCG27585, isoform CRA_b |
| 43 | 149017737 | rCG53077 |
| 44 | 149039655 | rCG57366 |
| 45 | 1838924 | regucalcin |
| 46 | 32563565 | Serine protease inhibitor |
| 47 | 149053083 | similar to novel protein of unknown function (DUF423) family member (predicted), isoform CRA_e |
| 48 | 149053425 | tyrosine 3-monooxygenase/tryptophan 5-monooxygenase activation protein, epsilon polypeptide, isoform |
| 49 | 56854 | unnamed protein product |
| 50 | 6561272 | uromodulin |
| 51 | 149036532 | actin, gamma 2, isoform CRA_b |
| 52 | 149056723 | apolipoprotein E, isoform CRA_e |
| 53 | 56847618 | keratin, type I cytoskeletal 16 |
| 54 | 207042 | serine protease inhibitor 1 |
| 55 | 347800746 | serine protease inhibitor A3K precursor |

| **Table S2-g: Proteins common to male and female lepto rat urine exosomes: absent in control animals.** | | |
| --- | --- | --- |
| **Sl #** | **Gene Id** | **Name of the protein** |
| 1 | 149057276 | alanyl (membrane) aminopeptidase |
| 2 | 47087113 | choline transporter-like protein 4 |
| 3 | 13786200 | voltage-dependent anion-selective channel protein 1 |
