# Supplementary material for: Proteomic Analysis of Urine Exosomes Reveals Renal Tubule Response to Leptospiral Colonization in Experimentally Infected Rats
Source: PLoS Negl Trop Dis. 2015 Mar 20;9(3):e0003640. doi: 10.1371/journal.pntd.0003640 (PMC4368819; doi:10.1371/journal.pntd.0003640)
Supplement: S3 Table — (DOCX) [file pntd.0003640.s008.docx]

| **Table S3 Raw data of *Leptospira* qPCR of rat urine** | | |
| --- | --- | --- |
| **Serial #** | ***Leptospira* copy #** | **time after infection** |
| **4a** | 0 | 7 days |
| **4b** | 3113.43 | 14 days |
| **4c** | 0 | 21 days |
| **4d** | 0 | 28 days |
| **4e** | 24990.9 | 29 days |
|  |  |  |
| **5a** | 746511.19 | 7 days |
| **5b** | 1983217.97 | 14 days |
| **5c** | 4204984.26 | 21 days |
| **5d** | 3923534.99 | 28 days |
| **5e** | 14741299.5 | 29 days |
|  |  |  |
| **6a** | 0 | 7 days |
| **6b** | 0 | 14 days |
| **6c** | 0 | 21 days |
| **6d** | 0 | 28 days |
| **6e** | 0 | 29 days |
|  |  |  |
| **7a** | 172146.88 | 7 days |
| **7b** | 1330446.13 | 14 days |
| **7c** | 1611900.65 | 21 days |
| **7d** | 3687257.6 | 28 days |
| **7e** | 1006869.3 | 29 days |
|  |  |  |
| **8a** | 716993.94 | 7 days |
| **8b** | 0 | 14 days |
| **8c** | 0 | 21 days |
| **8d** | 0 | 28 days |
| **8e** | 0 | 29 days |
|  |  |  |
| **9a** | 0 | 7 days |
| **9b** | 11831499.4 | 14 days |
| **9c** | 27518704.2 | 21 days |
| **9d** | 16860856.3 | 28 days |
| **9e** | 61209919.2 | 29 days |
